# The SRC-family serves as a therapeutic target in triple negative breast cancer with acquired resistance to chemotherapy

**DOI:** 10.1038/s41416-024-02875-5

**Published:** 2024-10-10

**Authors:** Eivind Valen Egeland, Kotryna Seip, Eleni Skourti, Geir Frode Øy, Solveig J. Pettersen, Abhilash D. Pandya, Maria A. Dahle, Mads H. Haugen, Alexander Kristian, Sigve Nakken, Olav Engebraaten, Gunhild M. Mælandsmo, Lina Prasmickaite

**Affiliations:** 1 Department of Tumor Biology, Institute for Cancer Research, The Norwegian Radium Hospital, Oslo University Hospital, Oslo, Norway; 2 Insitute for Clinical Medicine, University of Oslo, Oslo, Norway; 3 Department of Research and Innovation, Vestre Viken Hospital Trust, Drammen, Norway; 4 Centre for Cancer Cell Reprogramming, Institute of Clinical Medicine, Faculty of Medicine, University of Oslo, Oslo, Norway; 5 Centre for Bioinformatics, Department of Informatics, University of Oslo, Oslo, Norway; 6 Department of Oncology, Oslo University Hospital, Oslo, Norway; 7 Department of Medical Biology, Faculty of Health Sciences, The Arctic University of Norway-University of Tromsø, Tromsø, Norway

**Keywords:** Breast cancer, Cancer models, Tumour biomarkers, Cancer therapy

## Abstract

**Background:**

Resistance to chemotherapy, combined with heterogeneity among resistant tumors, represents a significant challenge in the clinical management of triple negative breast cancer (TNBC). By dissecting molecular pathways associated with treatment resistance, we sought to define patient sub-groups and actionable targets for next-line treatment.

**Methods:**

Bulk RNA sequencing and reverse phase protein array profiling were performed on isogenic patient-derived xenografts (PDX) representing paclitaxel-sensitive and -resistant tumors. Pathways identified as upregulated in the resistant model were further explored as targets in PDX explants. Their clinical relevance was assessed in two distinct patient cohorts (NeoAva and MET500).

**Results:**

Increased activity in signaling pathways involving SRC-family kinases (SFKs)- and MAPK/ERK was found in treatment resistant PDX, with targeted inhibitors being significantly more potent in resistant tumors. Up-regulation of SFKs- and MAPK/ERK-pathways was also detected in a sub-group of chemoresistant patients after neoadjuvant treatment. Furthermore, *High* SFK expression (of either *SRC, FYN* and/or *YES1*) was detected in metastatic lesions of TNBC patients with fast progressing disease (median disease-free interval 27 *vs* 105 months).

**Conclusions:**

Upregulation of SFK-signaling is found in a subset of chemoresistant tumors and is persistent in metastatic lesions. Based on pre-clinical results, these patients may respond favorably to treatment targeting SFKs.

## Background

Triple negative breast cancer (TNBC) accounts for 15-20% of all breast cancers (BC) and represents the subgroup with the least favorable outcome [1]. Compared to other subgroups, TNBC has higher risk of early relapse, increased likelihood of distant metastasis and shorter overall survival [2]. Chemotherapy has until recently been the only treatment option [1], in which anthracycline/taxane-based regimens, preferentially as neoadjuvant treatment (NAT), has been a standard approach for patients with primary TNBC [3]. With recent improvements in the regimens used, approximately half of the treated patients show pathological complete response (pCR), which is associated with prolonged survival [4, 5]. However, patients with residual disease experience increased likelihood of recurrence and the development of distant metastases [6, 7]. Thus, resistance to chemotherapy and subsequent relapse represents a significant challenge in the clinical management of TNBC in both the curative and metastatic setting, and emphasize the need for new therapeutic opportunities [8].

Despite being grouped as a single disease, TNBC is biologically and clinically highly heterogeneous, which creates additional challenge when searching for better therapeutic options. Considerable efforts have been made over the last decade to sub-classify TNBC based on molecular profiling [9–11]. Lehmann et al. [9, 12]. identified initially six, later revised to four, subtypes with characteristic molecular patterns, distinct prognosis, and differential response to standard-of-care chemotherapy. A number of analogous studies attempted to sub-classify TNBC combining various -omics approaches [10, 13, 14], and despite overlap, a large variability in classification has been observed. Based on the distinct molecular features, subtype-specific actionable targets have been proposed for individualized therapy [14–16]. Furthermore, it has been observed that TNBC patients with residual disease after NAT, frequently experience a change in the molecular subtype [17]. This suggests that treatment-associated molecular alterations in patients without pCR might have implications for the optimal choice of adjuvant therapy.

The extensive inter-tumoral heterogeneity in TNBC per se, and in response to treatment, makes identification of actionable molecular patterns challenging in clinical cohorts. Pairs of tumors with the same genetic background but distinct chemo-sensitivity can facilitate detection of resistance-associated molecular traits. Such pairs can be generated in patient-derived xenografts (PDXs) that are established by implanting patient tumor pieces into immunocompromised mice and represent useful tools for precision oncology, including investigation of biomarker-driven treatment options [18]. PDXs generally retain fidelity to the originating tumors both with respect to the main genomic aberrations and gene expression profiles, recapitulating intrinsic subtypes of BC [19, 20]. By exposing PDXs to clinically relevant treatments, tumors with features of resistance can be generated [21]. In the current study, we use an isogenic pair of PDXs consisting of paclitaxel-sensitive and -resistant variants. It enables identification of molecular traits linked with chemoresistance, which in turn can aid in sub-grouping TNBC patients and pinpointing candidates for next-line treatment.

## Methods

### PDXs maintenance and treatment in vivo

All in vivo experiments were performed using female Hsd: Athymic Nude-*Foxn1nu* mice, locally bred at the Department of Comparative Medicine at the Norwegian Radium Hospital, Oslo University Hospital (OUH, Oslo, Norway). MAS98.12 PDX was established in-house as described previously [22] and maintained by serial passaging, implanting 2–3 mm^3^ pieces of the tumor under the skin above the thoracic mammary glands of 6-8 week-old mice. Tumor growth was followed by measuring their size (length, L and width, W) using a caliper, and the tumor volume was calculated as W^2^ × L × 0.5. Implanted tumors with no growth at treatment initiation were excluded. Only tumors that reached a priori decided volume *i.e*. palpable (on average 40–60 mm^3^) and less than 200 mm^3^, were included. Treatment was initiated when tumor volume reached 60–200 mm^3^. All treatments were given in a volume corresponding to 10 µl/g body weight, and for all treatment groups at least five animals were included based on previous experience with the MAS98.12 model [23, 24]. Animals sacrificed due to off-treatment effects, thus not receiving a full treatment regimen, were excluded. Animals were randomized into distinct groups to ensure average tumor volume to be similar across treatment groups at the start of treatment. To minimize potential confounders, the order of treatment and animal/cage location was random, and data analyst was not aware of grouping until experiments had been finalized. All tumors receiving treatment were included in the analyzes. Relative tumor volume was calculated by normalizing to the volume at the day when treatment was started. Assessment of the treatment effect on tumor growth was performed by calculating area under the curve (AUC) for each tumor by using GraphPad Prism and calculating relative AUC with respect to the untreated controls. Treatment toxicity was monitored by following body weight and body condition. At experimental endpoint, the mice were sacrificed by cervical dislocation. Tumors were excised and snap frozen in liquid nitrogen for subsequent molecular analyzes.

The following drugs were used for treatment in vivo: paclitaxel (Mylan Laboratories Inc/Viatris), eribulin (Eisai AB, Sweden), docetaxel (Eurasia’s Chemicals and API, Mumbai, India), cabazitaxel (Biochempartner Co. Ltd., China) and carboplatin, doxorubicin, capecitabine (all from Accord Health Care, Solna, Sweden). Paclitaxel, eribulin, carboplatin and doxorubicin were diluted in 0.9% saline, docetaxel and cabazitaxel were diluted in 0.9% saline w/0.6% ethanol, while capecitabine tablets (150 mg) were dissolved in 40 mM citric-buffer w/5% Acacia gummi. All taxanes and doxorubicin were given intravenously, carboplatin was given intraperitoneally, while capecitabine was applied per oral.

### PDX tissue cultures (PDXC) and treatment ex vivo

Resected tumors were processed and cultured as described previously [25]. Briefly, the minced tumors were digested with 2 mg/mL collagenase IV and 100 µg/mL DNase (both from Sigma-Aldrich, St.Louis, MO, USA) for 30 min on rotation. After multiple centrifugations, the pellet containing the tissue fragments was re-suspended in BC organoid medium (OM + , specified in Supplementary Table S1) and plated in low adhesion plates. After 2–3 days, the tissue suspension was filtered through a 100 µm cell strainer and left to sediment for 2–5 min. The fragment-enriched pellet was resuspended in OM+ to a concentration of 7 - 9 fragments/µL. After addition of 30% Matrigel (Corning, New York, USA), droplets of 10 µL fragment/Matrigel mix were added to 96-well plates. The domes were allowed to solidify at 37 °C for 30 min before addition of 90 µL of OM + . The next day, 100 µL of OM+ with the desired concentration of the drug was added. The following drugs were used: saracatinib (AZD0530), dasatinib, cobimetinib (GDC-0973) and capivasertib (AZD5363) (all from Selleckchem, Houston, TX). After six days of treatment, the effect was scored by measuring metabolic activity in the PDXCs by CellTiter-Glo3D assay (Promega, Madison, WI, USA) following the manufacturer’s protocol.

### RNA sequencing

#### RNA isolation and sequencing

Total RNA was isolated from snap frozen tissue samples dissolved in 600 µl RLT Plus buffer w/2 mM DTT (Qiagen, Hilden, Germany) using TissueLyser LT (Qiagen) for 2×4 minutes at 30 Hz. Homogenized lysate was passed through a QIAshredder (Qiagen) spin column at 20.000 g for 30 seconds to remove debris, before total RNA was extracted by QIAcube Connect (Qiagen) with an AllPrep® DNA/RNA/miRNA Universal kit (Qiagen) according to manufacturer’s protocol. Finally, RNA was quantified on a Quibit Fluorometer (Thermo Fisher Scientific, Waltham, MA, USA) with the QubitTM RNA High Sensitivity Assay kit (Thermo Fisher Scientific), and RNA integrity number (RIN) determined on a 2100 Bioanalyzer (Agilent, Santa Clara, CA, USA) with the Agilent RNA 6000 Pico Kit (Agilent).

Sequencing libraries were prepared with KAPA RNA HyperPrep Kit (Roche, Basel, Switzerland) followed by Twist Human Comprehensive Exome (Twist Bioscience, South San Francisco, CA, USA) for mRNA capture, according to the respective manufacturer’s protocol. Samples were sequenced with Illumina NovaSeq6000 reagent kit v1.5 (Illumina, San Diego, CA, USA) on Illumina NovaSeq6000 or Illumina NextSeq500 with Illumina NextSeq500/550 reagent kit v2.5 at the Genomics Core Facility, Institute for Cancer Research, OUH, generating pair-end reads between 75–151 bp read length.

RNAseq data processing and analyzes are given in detail in Supplementary methods.

### Protein-protein interaction networks

Protein-protein interaction (PPI) networks were generated by selecting the 400 genes with highest absolute log2 fold change (LFC), among significant genes (padj < 0.05) from the bulk RNAseq differentially expressed gene (DEG) analyzes. This list of genes was uploaded to STRING (v11.5; https://version-11-5.string-db.org/ [26]) where protein–protein interactions were determined with the highest confidence setting (0.9). Networks were exported to Cytoscape [27], where genes with >2 edges were kept for the final interaction map.

### Preparation of protein lysates

For PDX, mechanically minced cryopreserved tissue (approximately 30 mg) was placed in 750 µL ice-cold lysis buffer (1% Triton X-100, 50 mM HEPES pH 7.4, 150 mM NaCl, 1.5 mM MgCl2, 1 mM EGTA, 100 mM NaF, 10 mM Na pyrophosphate, 1 mM Na3VO4, 10% glycerol) and 1 × pStop/cOmplete (Roche Applied Science, Mannheim, Germany), then lysed using Precellys lysing kit and Precellys tissue homogenizer (Bertin Technology, Montigny-le-Bretonneux, France). For the lysates used in the RPPA analysis, protein concentration was adjusted to 1.5 µg/mL, mixed with 4xSDS Sample Buffer (40% Glycerol, 8% SDS, 0.25 M Tris-HCl, pH 6.8) and denatured for 5 min at 95 ^o^C.

### Proteomic analyzes by Reverse-Phase Protein Array (RPPA)

The RPPA profiling was performed at the RPPA core facility of MD Anderson Cancer Center (Houston, TX) as previously described [28]. Briefly, serial diluted protein lysates were arrayed onto nitrocellulose-coated slides (Grace Bio-labs, Bend, OR, USA) using an Aushon 2470 Arrayer (Aushon BioSystems, Billerica, MA, USA), including the spots corresponding to positive and negative controls prepared from mixed-cell lysates and dilution buffer, respectively. Each slide was probed with a validated primary antibody plus a biotin-conjugated secondary antibody.

Prior to data normalization, mouse antibodies were removed to avoid unspecific signal, thus, 429 proteins, including 92 phosphorylated proteins, were included for normalization. Level 3 data were obtained for downstream analyzes by first log2 transforming raw data quantified with RPPA SPACE [29], before bidirectional normalization by median-centering first by antibody then by sample. Differential expressions were determined by the R package *limma*, by fitting multiple linear regression model with the function *lmFit*.

Pathway activity score (PAS) was defined for each sample as the sum of the protein expression levels of positive regulators minus that of negative regulators in a particular pathway and was calculated as previously described [30]. The proteins predictive for MAPK/ERK and PI3K/AKT pathway activity were predefined [30], while the proteins used to calculate the SRC-Family Kinases (SFKs) PAS were defined based on literature search (specified in Supplementary Table S2).

### Simple Western Immunoassay (SWI)

Proteins of interest were analyzed by SWI using a PeggySue^TM^ instrument (ProteinSimple, San Jose, CA). Protein lysate concentration was adjusted to 0.8 µg/µL, and protein separation was performed by using 12–230 kDa size separation master kit (SM-S001; ProteinSimple) according to the manufacturer’s instructions. Primary antibody incubation time was set to 60 min, while other settings were kept on default. The Compass software (Protein Simple) was used to control the instrument and analyze the data. The antibodies and their dilutions are specified in Supplementary Table S3. For all proteins of interest, the expression (area under the curve) was normalized to the expression of the loading controls (GAPDH or β-ACTIN) in each sample. Detection curves for all samples used in this study are given in Supplementary Fig. S11-S13).

### Clinical cohorts

#### NeoAva

From the NeoAva phase II clinical trial (NCT00773695), TNBC samples collected before NAT at screening (NASC) and post-NAT at surgery (NA25) for patients without pCR were analyzed for gene and protein expression as described previously in [31] and [32], respectively. All transcriptomics data was retrieved from Silwal-Pandit et al. [31]. RPPA protein expression data at NASC was obtained from Haugen et al. [32], while the data at NA25 was retrieved from an ongoing study and normalized together with the NASC data using the method described by Haugen et al. [32].

#### MET500

Transcriptomics data from metastatic lesions in the MET500 cohort of solid cancers [33], was retrieved from https://xenabrowser.net, while clinical data was taken from Supplementary Table S1 in Robinson et al. [33]. The unique TNBC samples (*n* = 40) and the corresponding TNBC subtypes determined for these samples by Lehmann et al. [16], were used in this study. Gene expression data was prepared by log2-transforming *fkpm* values, and either used directly for survival analyzes, or median centered for use in heatmaps. Immune fraction generated by xCell [34] were retrieved from Supplementary Table S2 in Lehman et al. [16].

### Survival analyzes

Survival analyzes were performed in R with functions *survfit* and *ggsurvplot*, from packages *survival* (v3.5-7) and *survminer* (v0.4.9), respectively. *P*-values were calculated for each curve by the log-rank method.

### Presentation and statistics

Data processing, analyzes and figure generation were done in *R* (v4.3.1) programming language with *RStudio* (2023.09.0 + 463). Figures are generated with the R package *ggplot2* (v3.4.4) unless indicated otherwise. Statistics were performed using *rstatix* (v0.7.2), while Pearson correlation was done with base R functions *cor* and *cor_test*. Pairwise comparisons were performed for relevant groups as indicated using a two-sided t-test with Welch’s correction of unequal variance. If data was determined not to be normally distributed by the Shapiro-Wilks test, a two-sided Mann–Whitney U test was used instead. For both tests, a *p* < 0.05 was considered statistically significant, and labeled with an asterisk (*) in figures.

## Results

### Generation of an isogenic PDX pair with distinct sensitivity to taxanes

MAS98.12 PDX has been established in-house, and as shown previously, recapitulates the main histological and genomic features of the parental patient tumor [22]. Whole exome sequencing (Supplementary Table S4) validated the presence of TP53 mutation in codon 120, also detected in the patient tumor biopsy [22].

**Fig. 1:**
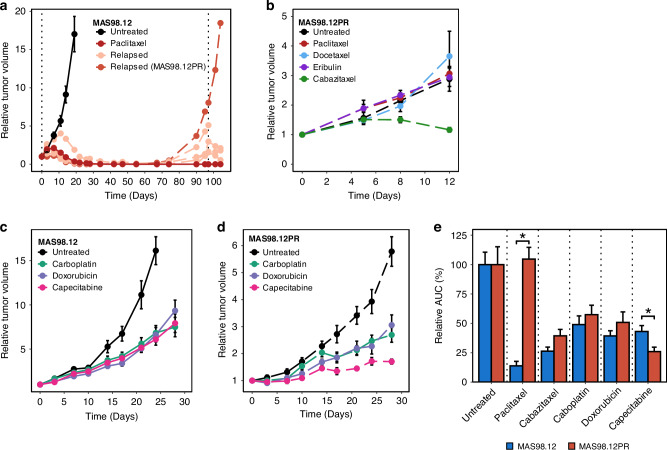
Chemosensitivity of MAS98.12 and MAS98.12PR PDX. **a** Relative tumor volume of MAS98.12 treated with paclitaxel at a concentration of 15 mg/kg twice/week or 30 mg/kg once/week for three weeks (untreated *n* = 11, paclitaxel *n* = 19, where 5 relapsed). The individual relapsed tumors (indicated by dashed lines) were exposed to the original dose of paclitaxel, and one of them did not show any response, becoming the origin of the resistant sub-line MAS98.12PR. **b** Relative tumor volume of MAS98.12PR treated with taxanes: 10 mg/kg paclitaxel (*n* = 8), 6 mg/kg docetaxel (*n* = 6), 0.5 mg/kg eribulin (*n* = 8) and 15 mg/kg cabazitaxel (*n* = 7) applied twice per week for two weeks; untreated *n* = 7. **c**, **d** Relative tumor volume of MAS98.12 (**c**) and MAS98.12PR (**d**) treated with different chemotherapeutic agents: 50 mg/kg carboplatin once per week (*n* = 9/12), 8 mg/kg doxorubicin once per week (*n* = 9/11) and 755 mg/kg capecitabine five times per week (*n* = 9/9) for three weeks. The curves in **a**–**d** represent mean ± SEM; **e** Efficacy of the treatment (specified in **b**–**d** in MAS98.12 and MAS98.12PR presented as relative AUC (compared to the untreated controls) on week two (for drugs in **b**) or three (for drugs in **c**, **d**); mean ± SEM; *p < 0.05.

MAS98.12 PDX showed high sensitivity to paclitaxel, a microtubule-targeting agent used as standard treatment for patients with TNBC. After three weeks on-treatment, all nineteen tumors decreased in size (Fig. 1a and Supplementary Fig. S1A). However, after ten weeks, five tumors relapsed, with one not responding to the repeated treatment with paclitaxel (Fig. 1a and Supplementary Fig. S1A). This non-responding tumor was the origin of the paclitaxel-resistant sub-line MAS98.12PR, which also at later generations showed complete insensitivity to paclitaxel (Fig. 1b and Supplementary Fig. S1B). Furthermore, MAS98.12PR also showed lack of sensitivity to other microtubule-targeting agents, docetaxel and eribulin, but not cabazitaxel, which inhibited tumor growth similarly to what was observed in MAS98.12 (Fig. 1b and Supplementary Fig. S1B,C). Compared to MAS98.12, MAS98.12PR showed no difference in sensitivity to the DNA-targeting chemotherapeutics, carboplatin and doxorubicin (Fig. 1c,d), which reduced the tumor growth by approximately 50% (Fig. 1e). MAS98.12PR, however, displayed a slightly better response to capecitabine, a precursor of 5-FU (Fig. 1c,d), which induced approximately 70% reduction in tumor growth compared to 50% in MAS98.12 (Fig. 1e). In subsequent figures, MAS98.12 and MAS98.12PR are abbreviated as PS (paclitaxel-sensitive) and PR (paclitaxel-resistant), respectively.

Immunohistochemical comparison of MAS98.12PR with MAS98.12 (Supplementary Fig. S2) revealed equally high positivity in the epithelial markers (EPCAM, CK19 and E-CADHERIN), slightly stronger staining for the mesenchymal marker (VIMENTIN) and a slightly weaker staining for the proliferation marker Ki-67. In contrast to MAS98.12, MAS98.12PR stained positive for the multidrug resistance protein 1 (MDR1), a well described mediator of paclitaxel resistance [35].

A comparison of genomic aberrations (point mutations and short insertions/deletions) in MAS98.12PR *versus* MAS98.12 revealed a slight increase in overall mutational burden (Supplementary Fig. S2A) and several aberrations specific to MAS98.12PR (Supplementary Table S5). While most variants were of uncertain significance (VUS), *ATRX* (alpha-thalassemia mental retardation X-linked) was predicted as likely oncogenic, though at low allelic fraction (VAF) (Supplementary Fig. S2B). Notably, ATRX is a tumor suppressor that functions as a “guardian” of genome stability [36], and inactivation of its function due to mutations may potentially explain the observed increase in mutational burden in MAS98.12PR.

In conclusion, we have generated a paclitaxel-resistant PDX, which together with the sensitive counterpart constitutes an isogenic pair for studies on molecular alterations associated with resistance.

### Large transcriptional changes are associated with acquisition of chemoresistance

**Fig. 2:**
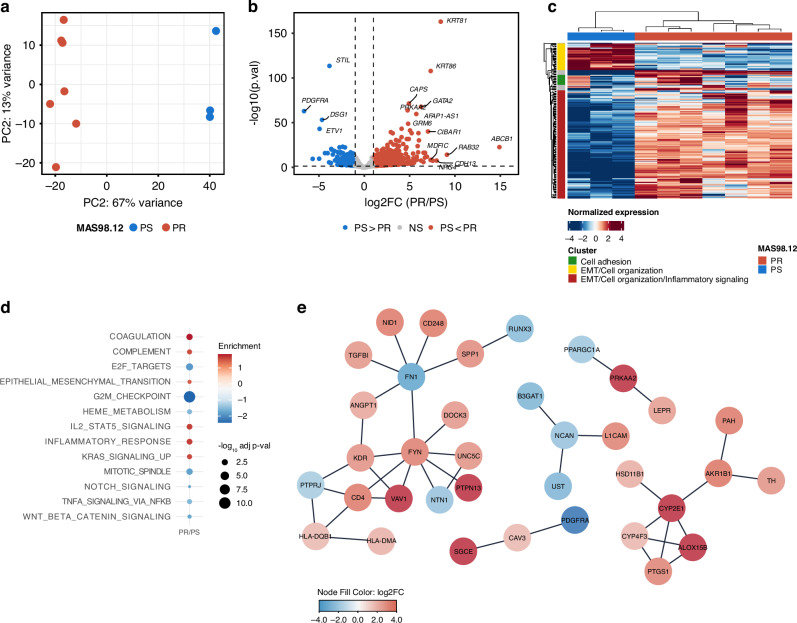
Transcriptional changes in MAS98.12PR compared to MAS98.12. **a** PCA of top 500 genes based on expression variance in RNAseq samples from PS (*n* = 3) and PR (*n* = 7). **b** Volcano plot of DEGs. Dotted line indicates padj = 0.05 and |LFC | > 1. **c** Heatmap of the 200 genes with highest variation across all samples. Three distinct clusters are highlighted with features defined by functional characteristics overlapping with genes sets in the Hallmark and KEGG signature databases. **d** GSEA performed on the Hallmark Signature Database, on values ranked by LFC from PR/PS. **e** PPI network generated with top 400 DEGs by using the STRING database, and the highest confidence (0.9) to select predicted interactions. Genes with >2 edges are shown.

To map the molecular traits distinguishing MAS98.12PR from MAS98.12, transcriptome profiles were generated by bulk RNAseq. Principal component analysis (PCA) of the 500 genes with highest variation in the RNAseq data, revealed strong separation between the MAS98.12 and the MAS98.12PR samples (Fig. 2a), illustrating that acquired resistance is accompanied by global changes in the transcriptome. In line with this observation, differential expression analyzes revealed more than 4000 genes to be significantly altered between the models. While approximately the same number of genes were found to be up- and down-regulated, a general stronger induction of the up-regulated genes was observed (738 genes with LFC > 1 *versus* 259 with LFC < −1). The *ABCB1* transcript encoding MDR1 showed the highest induction with approximately 15 LFC (Fig. 2b). Clustering of the 200 genes with the highest variation across all samples identified three distinct clusters, characterized by genes involved in EMT, Cell organization, Cell adhesion and Inflammatory signaling (Fig. 2c). Similarly, GSEA on genes ranked by LFC was performed to identify biological pathways significantly altered in the resistant tumors. By applying GSEA to the Hallmark gene sets, immunity-related processes (inflammatory response, IL2-STAT5 signaling, complement) as well as EMT and KRAS signaling were identified among enriched pathways in the resistant tumors (Fig. 2d). Down-regulated pathways were dominated by cell cycle associated processes (G2M checkpoint, Mitotic spindle, and E2F-targets), in line with the well characterized effects of taxanes. Similar trends were observed when applying GSEA to the KEGG and GO Biological Pathway databases (Supplementary Fig. S4A,B). Altogether, this indicates that multiple biological processes were affected upon acquisition of paclitaxel resistance.

To determine central molecules in the resistant phenotype, predicted protein-protein interaction (PPI) networks, based on the 400 most significant differentially expressed genes, was generated. By choosing a stringent confidence (0.9 in the STRING database) only well-established PPIs were assessed. By defining hubs of genes as having >2 interactions (edges) with other genes in the list, *FYN* (LFC 3.5; padj < 2.11 × 10^−20^) was detected as a central hub in the network (Fig. 2e). FYN is one of eight members in the SRC-Family Kinases (SFKs), which share similar funtions in transducing multiple oncogenic signals [37, 38].

### Proteome analyzes reveal candidate targets in chemoresistant tumors

**Fig. 3:**
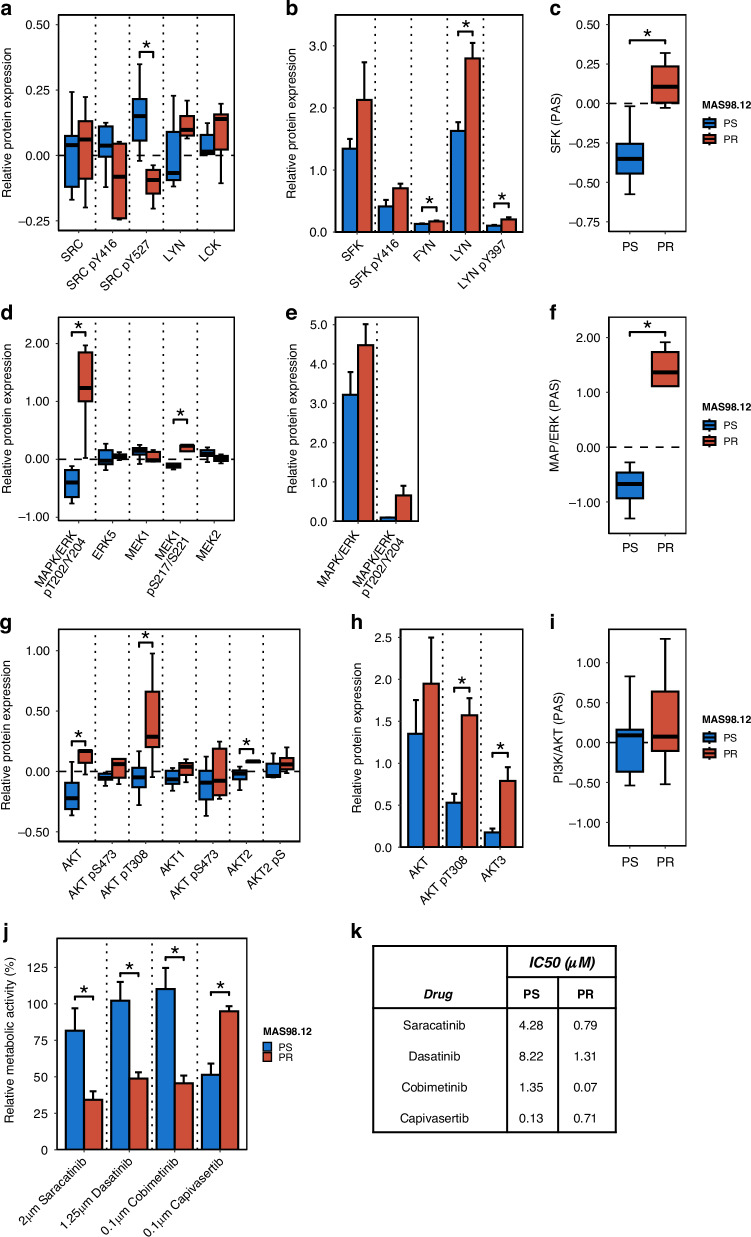
Involvement of SFK-, MAPK/ERK- and PI3K/AKT-signaling pathways in MAS98.12PR compared to MAS98.12. Comparison of expression levels of (phospho)proteins from the SFK (**a**,**b**), MAPK/ERK (**d**,**e**) and PI3K/AKT (**g**,**h**) signaling pathways in PDXs as detected by RPPA (**a**, **d**, **g**, *n* = 7/5) and validated by SWI (**b**, **e**, **h**, *n* = 3/5). PAS for SFK (**c**), MAPK/ERK (**f**) and PI3K/AKT (**i**) calculated from the RPPA data. For **a**,**d** and **g**, *p*-values were retrieved from differential analysis with *limma*. **j** Sensitivity of MAS98.12 and MAS98.12PR tissue to saracatinib, dasatinib, cobimetinib and capivasertib as assessed in PDXCs after treatment for six days; the effect was evaluated by measuring metabolic activity normalized to the corresponding untreated controls (mean ± SEM; *n* = 5/4/6/3). *p < 0.05. **k** IC50 values (µM) of drugs specified in **j** in PDXCs from MAS98.12 and MAS98.12PR.

To further explore whether paclitaxel resistant tumors have activated SFK-signaling, proteomic analysis by RPPA was performed. By comparing MAS98.12PR to MAS98.12, SRC pY527, which leads to autoinhibition of the pathway [37], was noted among the most down-regulated proteins (Fig. 3a, Supplementary Fig. S5A and Supplementary Table S6). To validate the activation of the SFK pathway, multiple SFKs (SRC, FYN and LYN), and the activating phosphorylations (SFK pY416 and LYN pY397), were analyzed by SWI. In line with the RPPA data, higher levels of these proteins were detected in the MAS98.12PR tumors (Fig. 3b), although intra-tumoral heterogeneity, as revealed by SFK pY416 IHC, was observed (Supplementary Fig. S5B). In addition, the overall activity of the SFK pathway in both PDX models was calculated using RPPA pathway activity score (PAS) as described by Akbani et al. [30]. The difference in SFK-PAS indicated that SFK-signaling was significantly more active in MAS98.12PR compared to MAS98.12 (Fig. 3c).

The RPPA analysis combined with SWI-validation also uncovered significant up-regulation of MAPK/ERK pT202/Y204 in MAS98.12PR (Fig. 3d,e, Supplementary Fig. S5A and Supplementary Table S6), suggesting activation of the MAPK/ERK signaling. This was further supported by the significantly higher MAPK/ERK-PAS in MAS98.12PR compared to MAS98.12 (Fig. 3f).

Furthermore, the RPPA and SWI data revealed significantly higher levels of pan-AKT and pan-AKT pT308 in MAS98.12PR (Fig. 3g,h and Supplementary Table S6), although the levels of the two main isoforms, AKT1 or AKT2, showed relatively small changes (Fig. 3g). When the level of the third isoform, AKT3 (not present on RPPA) was evaluated, its up-regulation in MAS98.12PR on both protein and gene level was revealed (Fig. 3h and Supplementary Fig. S4C). Despite elevation in AKT3, no significant difference in AKT-PAS could be observed between MAS98.12PR and MAS98.12 (Fig. 3i).

In conclusion, enhanced activity in SFKs- and MAPK/ERK- signaling were identified in paclitaxel-resistant tumors, suggesting these pathways as potential targets for therapy.

### Chemoresistant tumors are sensitive to SFK- and MAPK/ERK-pathway inhibitors

The effect of targeting SFKs- and MAPK/ERK- signaling was explored ex vivo using PDX cultures (PDXCs), which enable maintenance of viable BC tissue and preserve its proliferative capacity (Supplementary Fig. 6A) [25]. Previously, we [25] and others [39] have demonstrated strong correlation between tissue drug-sensitivity in PDXCs and the corresponding PDXs, arguing for PDXCs as a possible substitute for in vivo models. PDXCs from MAS98.12PR and MAS98.12 generally preserved the main molecular differences detected in the PDXs *i.e*. up-regulation of SFKs, MAPK/ERK pT202/204 and AKT3 in the resistant tumor tissue (Supplementary Fig. 6B).

To explore whether targeting of the up-regulated pathways affected tissue viability, the PDXCs were treated with the respective inhibitors. Compared to MAS98.12, MAS98.12PR tissue showed significantly higher sensitivity to the SFKs inhibitors, saracatibin and dasatinib (Fig. 3j,k and Supplementary Fig. S6C–G). Likewise, the MAS98.12PR tissue was more sensitive to the MAPK/ERK pathway inhibitor cobimetinib, which targets MEK (Fig. 3j,k and Supplementary Fig. S6H,I). In contrast, the MAS98.12PR tissue was less sensitive to the AKT inhibitor, capivasertib (Fig. 3j,k and Supplementary Fig. S5J). To confirm that saracatinib and cobimetinib inhibitied the SFK- and MAPK/ERK-pathways in PDXCs, the levels of the phosphorylated target proteins were assessed. Saracatinib reduced SFK pY416 levels, and cobimetinib diminished MAPK/ERK1/2 pT202/Y204 in both tissues (Supplementary Fig. S7), indicating a specific molecular response. Altogether, this data validated the functional significance of SFKs and MAPK/ERK pathways in paclitaxel-resistant tumors and proposed these pathways as actionable targets.

### SFK- and MAPK/ERK-signaling in chemoresistant patients

**Fig. 4:**
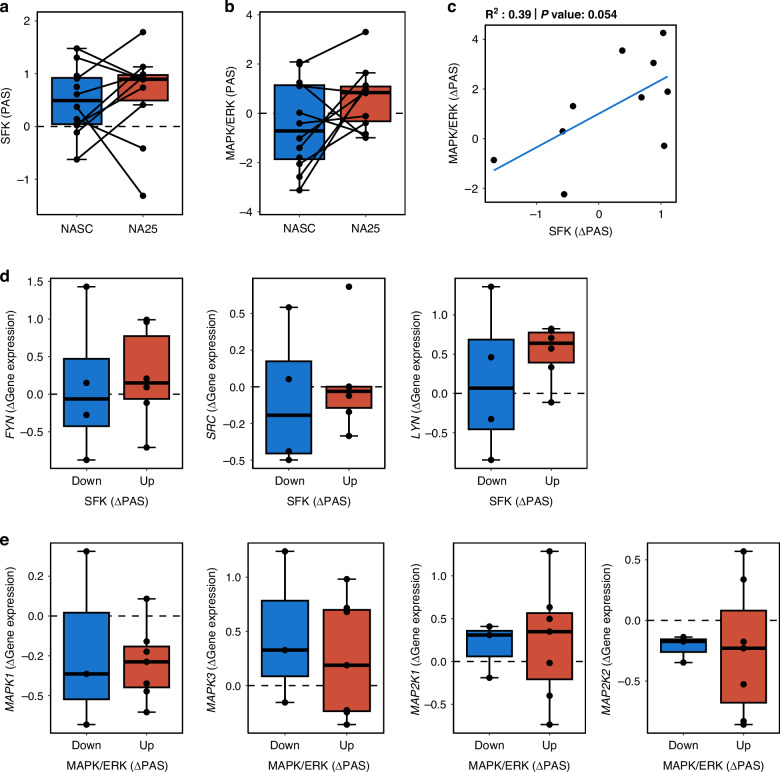
SFK- and MAPK/ERK-pathway activity in chemoresistant tumors from the NeoAva clinical study. PAS for SFK (**a**) and MAPK/ERK (**b**) calculated from RPPA data at screening (NASC; *n* = 12), and after NAT at week 25 (NA25; *n* = 10) in TNBC tumors without pCR. **c** Correlation plot of ∆PAS (NA25-NASC) between SFK and MAPK/ERK. **d** ∆Gene expression at NA25-NASC for SFK members in tumors with Up or Down ∆PAS SFK. **e** ∆Gene expression for MAPK/ERK members in tumors with Up or Down ∆PAS MAPK/ERK.

To assess the clinical relevance of the upregulated pathways, data from the neoadjuvant clinical trial NeoAva was utilized. Longitudinal samples from the TNBC patients without pCR to neoadjuvant chemotherapy were analyzed by RPPA. By comparing SFK- and MAPK/ERK-PAS in biopsies taken at surgery *versus* at screening, increased activity in both pathways was found for a subset of the patients (6/10 and 7/10, respectively, Fig. 4a,b), and a positive correlation in activation of the two pathways was observed, though marginally not significant (Fig. 4c). NAT-induced up-regulation in a subset of patients was also detected at the transcriptional level (Supplementary Fig. S8). The majority of tumors with up-regulated SFK-PAS upon NAT, also showed elevation in expression of SFK genes, *FYN* and *LYN (*Fig. 4d & Supplementary Fig. 8A), supporting the results from the protein PAS analysis and suggesting that changes in SFK pathway could potentially be predicted at the transcriptional level. In contrast, no correlation between changes in MAPK/ERK-PAS and transcription of associated genes was observed (Fig. 4e & Supplementary Fig. S8B), indicating the necessity of (phospho)proteome data for capturing alterations in MAPK/ERK signaling.

Altogether, this indicates NAT-associated elevation in SFK and MAPK/ERK signaling in a subgroup of the resistant patients, and that gene expression can be utilized to further explore SFK in patient cohorts.

### Expression of SFKs in subgroups of metastatic TNBC

Since TNBC without pCR are prone to metastasize, we hypothezised that the molecular traits disclosed in our chemoresistant model, are more prominent in metastatic rather than primary tumor lesions. To explore associations between SFKs and clinical outcome, the MET500 cohort of metastatic solid tumors was used, as this includes gene expression data from metastatic lesions and information on interval between primary disease and metastasis from 40 TNBC patients [33].

**Fig. 5:**
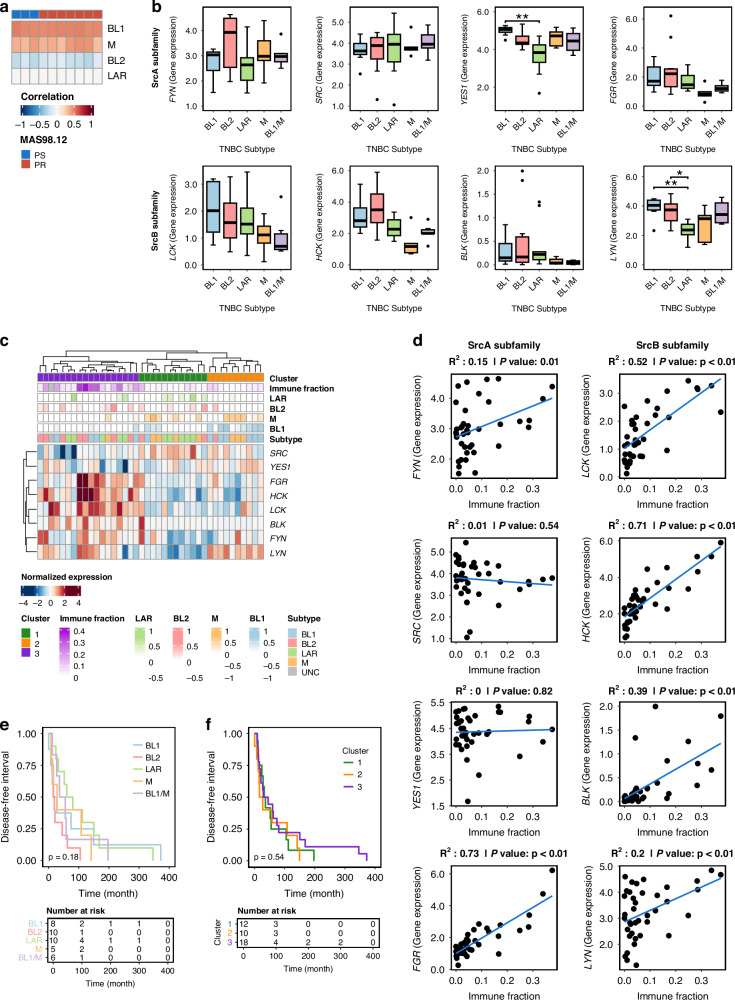
SFKs gene expression and association with TNBC subtypes in metastatic lesions from MET500 cohort. **a** Correlation with TNBC subtypes in MAS98.12 and MAS98.12PR. **b** Expression level of the eight SFK within the TNBC subtypes in the MET500 cohort. TNBC subtypes were defined as the 4-subtypes reported by Lehmann et al., in addition to samples showing dual correlation to both BL1 and M (BL1/M). **c** Hierarchical clustering of SFKs in samples from **b**, highlighting three distinct clusters as well as inferred immune fractions by xCell. **d** Correlation between SFKs expression and Immune fractions determined by xCell. **e** Kaplan–Meier plot showing disease-free interval for BL1/M compared to the established TNBC subtypes. **f** Disease-free interval for the three clusters defined in (**c**).

First, association between SFKs expression and TNBC subtypes as defined by Lehmann et al. [9, 12] was assessed. We hypothesized that the up-regulation of SFK-signaling could be a trait of the subtype represented by our PDX model. When MAS98.12 and MAS98.12PR PDX were classified according to Lehmann’s TNBC subtypes, they showed strong correlation with both BL1 and M subtypes, later referred to as BL1/M (Fig. 5a). Further, expression of all eight members of SFKs (SRC-A sub-family: *SRC, FYN, YES, FGR*; and SRC-B sub-family: *LYN, BLK, HCK, LCK*) were assessed according to the TNBC subtypes. The BL1/M tumors did not differ significantly from other subtypes with respect to expression of *SRC, FYN* and *LYN*, but showed a trend towards lower expression of *FGR* and the remaining members of the SRC-B sub-family (Fig. 5b). Next, hierarchical clustering defined three distinct clusters of SFK expression pattern (Fig. 5c). Cluster 1 and 2 included the majority of tumors correlating with BL1 and M. Cluster 3, on the other hand, included most of the BL2 tumors, and showed elevated levels of *FGR* and the SRC-B sub-family. Cluster 3 also displayed high immune fraction as inferred by xCell. Accordingly, and in line with the literature [40], strong correlation was found between the relative immune fraction and the levels of *FGR* and the SRC-B sub-family (except *LYN*) (Fig. 5d), suggesting their expression to be associated with the tumor immune microenvironment. In contrast, low/no correlation with immune fraction was found for either *SRC*, *FYN*, *YES1* or *LYN*, suggesting tumor intrinsic roles for these kinases. Despite the observed associations, there was no difference in disease-free interval between the tumors of BL1/M *versus* the other TNBC subtypes or the distinct SFK-clusters (Fig. 5e,f). Collectively, this suggested that SFK up-regulation is not limited to a particular TNBC subtype, and SFK-signaling could be a potential therapeutic target in metastatic TNBC irrespective of the subtype.

### High SFK expression in metastatic lesions defines patients with fast progressing disease

**Fig. 6:**
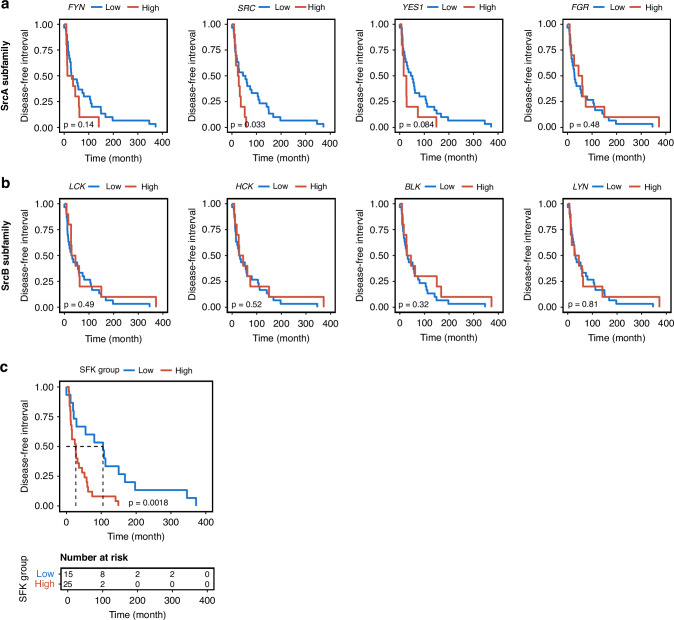
Expression of SRC-A sub-family members is associated with reduced disease-free interval in TNBC. Disease-free interval for the members of SRC-A (**a**) and SRC-B (**b**) sub-family in TNBC tumors from the MET500 cohort. High expression was defined by samples with expression >75% quantile for the respective SFK. **c** Disease-free interval for *High* expression of relevant SFK as >75% quantile for at least one of the ubiquitously expressed members (i.e. *SRC*, *FYN* or *YES1)* and the corresponding *Low* group.

To assess whether expression of the individual SFK members associates with disease-free interval in the MET500 cohort, *High* expression groups were defined as tumors in the 75% percentile for each of the SFKs. High expression of SFKs from the SRC-A subfamily correlated with shorter disease-free interval, in particular for *SRC*, but also *FYN* and *YES1* showed similar, but not significant, trends (Fig. 6a and Supplementary Fig. S9A). No such correlation was seen for any of the SRC-B subfamily members (Fig. 6b and Supplementary Fig. S9B). Therefore, stratification was redefined to *High* expression of at least one of three ubiquitously expressed SFKs *i.e. SRC, FYN* or *YES1*. This patient group (25/40) showed significantly shorter disease-free interval (Fig. 6c; median 27 *vs* 105 months; *p* = 0.002; Hazard Ratio = 3.2). Including *LYN* as a parameter for selecting tumors to the *High* group, did not improve the significance level between the *High* and *Low* groups (Supplementary Fig. S9C,D).

For comparison, no association was found between SFKs expression and overall or disease-specific survival in primary TNBC, when similar analysis was applied to the TCGA or METABRIC cohorts (Supplementary Fig. S10).

Altogether, this indicated that high levels of at least one of the three SFKs - *SRC, FYN or YES1* - define a sub-group of aggressive TNBC with fast metastatic progression.

## Discussion

In this study, we have revealed distinct molecular changes associated with treatment-induced resistance to paclitaxel. By using a patient-derived TNBC model we recapitulated recurrent disease after standard chemotherapy and identified signaling pathways that distinguish a sub-group of refractory tumors with fast metastatic progression. Finally, we proposed SFKs as candidate targets for next line treatment for this sub-group.

By -omics driven approaches, large overall changes were identified in the paclitaxel-resistant PDX, where the most significant alteration was up-regulation of the drug pump MDR1. The fact that MAS98.12PR was also resistant to docetaxel and eribulin (both can be transported by MDR1), but not cabazitaxel (not a substrate for MDR1), argues for MDR1 up-regulation as a major driver of paclitaxel-resistance in our model. Even though MDR1 is a well-established mechanism of chemoresistance, multiple attempts to exploit it as a therapeutic target have failed [41].

On the other hand, molecular alterations that develop as an adaptive response, along with development of chemoresistance, may uncover alternative targets for next-line treatment. We identified at least three SFKs (*SRC*, *FYN* and *LYN*) to be up-regulated, and constituting a central hub, in the paclitaxel-resistant PDX. This is in line with Kohale et al. [42], who detected activation of SFKs in PDXs with reduced sensitivity to paclitaxel. Up-regulation of SFKs was also found in a sub-group of resistant TNBC patients in the NeoAva clinical trial. Furthermore, elevated levels of SFKs were detected in the metastatic patients that experienced short disease-free interval in the MET500 cohort. Collectively, this suggests that up-regulated SFK-signaling is a feature of a subgroup of chemoresistant tumors, which in turn make them susceptible to SFK targeted therapy. In concordance, we demonstrated that the paclitaxel-resistant tumor tissue was more sensitive to the SFKs inhibitors, saracatinib and dasatinib. Likewise, Kohale et al. observed a correlation between SFK activity and sensitivity to dasatinib in vivo [42].

SFKs are well-established oncogenic kinases, which have frequently been implicated in tumorigenesis and metastasis, especially in BC [38]. In cohorts of primary TNBC (TCGA and METABRIC) we found no association between SFK levels and survival, while in metastatic TNBC from the MET500 cohort, a strong association between high SFKs levels and short disease-free interval was disclosed. This can be explained by the fact that the prevalence of SFK *High* tumors is low among primary TNBC without treatment [42] and that SFK up-regulation is provoked under therapy pressure. In line with the latter, NAT-associated up-regulation of SFKs in a sub-group of TNBC patients without pCR was observed in the NeoAva cohort, suggesting an adaptive response on-treatment. One could speculate whether a subset of relapsed tumors retain SFKs up-regulation after treatment withdrawal, to exploit this pathway for further progression into metastatic disease. The latter is in line with Zhang et al. [43], who demonstrated the significance of the activated SRC for BC metastasis, in particular to the bone. *High* SFK in MET500, however, is not strictly associated with bone metastasis as the cohort includes mostly lesions from other sites. Collectively, this suggests that SFKs are involved in multiple mechanisms promoting metastatic progression, and that SFK-dependent tumors are candidates for the targeted treatment irrespective of their TNBC subtype.

Due to the oncogenic role of SFKs and the proposed potential as a target, multiple clinical trials testing SFKs inhibitors have been performed in patients with metastatic BC (reviewed in [38]). However, SFK inhibitors, when used as single agents in unselected patient populations, have failed to improve patient outcomes [44, 45]. Combination of SFK inhibitors with other treatment modalities in unselected metastatic BC has shown some, but limited, clinical benefit [46, 47]. These studies together with the presented data and the study by Kohale et al. [42] emphasize the need for biomarkers to select SFK-dependent tumors that might benefit from the targeted treatment. Several attempts have been made to stratify metastatic BC patients for dasatinib treatment based on in vitro-derived *SRC*-gene signatures [48]. Unfortunately, these efforts have been unsuccessful. One recent study suggested that signature of tyrosine phosphorylation is a better predictor for selecting SFK-driven, dasatinib-sensitive PDXs, than transcript-derived signatures [42]. We have shown that high expression of at least one of the SRC-A subfamily members - specifically *SRC, FYN* or *YES1* - in metastatic lesions identifies patients with a short disease-free interval. It is tempting to speculate that this subgroup might benefit from SFK-targeted treatment, which remains to be validated in a relevant cohort. These three SFKs are known to be ubiquitously expressed and might execute tumor-intrinsic activity promoting metastasis and resistance (reviewed in [40, 49]). The expression of the remaining SFKs was not associated with disease-free interval but correlated with immune infiltration. This is in line with their known association with the immune microenvironment [40, 49].

Targeting SFKs has several challenges. One of them relates to SFKs themselves, as the family includes multiple members sharing similar structure and functions [37]. Based on our data, over-expression of one of three members is sufficient to identify fast-progressing, metastatic tumors, possibly due to hyper-activation of the signaling. Therefore, targeting should be able to affect multiple members of the family to avoid compensatory activation of the pathway. Another challenge links to the targeted drugs as clinically used SFK inhibitors (dasatinib, saracatinib, bosutinib) are not specific and inhibit multiple kinases not restricted to SFKs (reviewed in [38]). Of note, the recently developed selective SRC inhibitor eCF506 shows increased anti-tumor activity in TNBC models in vivo [50].

The MAS98.12PR PDX model revealed several other molecular alterations that could have therapeutic relevance. It was detected up-regulation of MAPK/ERK signaling along with enhanced sensitivity to the MEK inhibitor cobimetinib, suggesting the functional significance of this pathway in chemoresistant tumors. Elevation in MAPK/ERK pathway activity might be related to up-regulation of SFKs, which are known to facilitate the downstream signaling via MAPK/ERK [51]. In concordance with this, correlation between NAT-mediated up-regulation of both pathways was observed in a sub-group of the resistant NeoAva patients. Since MAPK/ERK-related changes were detected primarily at the protein level, their clinical relevance remains to be validated in cohorts with proteome data available.

Paclitaxel-resistant tumors also showed distinct up-regulation of AKT3, but with no indication of general activation of the PI3K/AKT pathway and response to the pan-AKT inhibitor capivasertib. Although we have not elucidated the mechanisms behind such an effect, it has previously been reported that AKT3 is particularly important for growth of TNBC, and that this isoform can confer resistance to pan-AKT inhibitors [52].

In conclusion, we have identified that chemoresistant, metastatic TNBC with fast-progressing disease demonstrate elevated expression of SFKs. Data from the PDX models implies that such tumors are sensitive to SFK targeting and motivate its further evaluation. A potential strategy for future clinical trials involves evaluating the expression of SRC-A sub-family members in post- *versus* pre-NAT biopsies to identify tumors with up-regulation. Subsequent ex vivo assessment of drug sensitivity in tumors with upregulated SFK could then facilitate the selection of patients who may benefit from targeted treatment.

## Supplementary information

Supplementary methods

Supplementary Figures

Supplementary Table S1

Supplementary Table S2

Supplementary Table S3

Supplementary Table S4

Supplementary Table S5

Supplementary Table S6

Supplementary Table S7

## Data Availability

All clinical material and data presented herein are available from the respective publications or upon request from the authors (see Methods section). RNAseq from MAS98.12 and MAS98.12PR are available at Gene Expression Omnibus (GSE265955), while normalized RPPA data are included as Supplementary Table S7. The PDX models MAS98.12 and MAS98.12PR are available upon reasonable request.

## References

[1] Bianchini G, Balko JM, Mayer IA, Sanders ME, Gianni L. Triple-negative breast cancer: challenges and opportunities of a heterogeneous disease. Nat Rev Clin Oncol. 2016;13:674–90. 27184417 10.1038/nrclinonc.2016.66 PMC5461122

[2] Dent R, Trudeau M, Pritchard KI, Hanna WM, Kahn HK, Sawka CA, et al. Triple-negative breast cancer: clinical features and patterns of recurrence. Clin Cancer Res. 2007;13:4429–34. 17671126 10.1158/1078-0432.CCR-06-3045

[3] Furlanetto J, Loibl S. Optimal systemic treatment for early triple-negative breast cancer. Breast care (Basel, Switz). 2020;15:217–26. 10.1159/000508759 PMC7383279 32774215

[4] Schmid P, Cortes J, Pusztai L, McArthur H, Kümmel S, Bergh J, et al. Pembrolizumab for early triple-negative breast cancer. N. Engl J Med. 2020;382:810–21. 32101663 10.1056/NEJMoa1910549

[5] Schmid P, Cortes J, Dent R, Pusztai L, McArthur H, Kümmel S, et al. Event-free survival with pembrolizumab in early triple-negative breast cancer. N. Engl J Med. 2022;386:556–67. 35139274 10.1056/NEJMoa2112651

[6] Cortazar P, Zhang L, Untch M, Mehta K, Costantino JP, Wolmark N, et al. Pathological complete response and long-term clinical benefit in breast cancer: the CTNeoBC pooled analysis. Lancet. 2014;384:164–72. 24529560 10.1016/S0140-6736(13)62422-8

[7] Liedtke C, Mazouni C, Hess KR, André F, Tordai A, Mejia JA, et al. Response to neoadjuvant therapy and long-term survival in patients with triple-negative breast cancer. J Clin Oncol. 2008;26:1275–81. 18250347 10.1200/JCO.2007.14.4147

[8] Bianchini G, De Angelis C, Licata L, Gianni L. Treatment landscape of triple-negative breast cancer — expanded options, evolving needs. Nat Rev Clin Oncol. 2022;19:91–113. 34754128 10.1038/s41571-021-00565-2

[9] Lehmann BD, Bauer JA, Chen X, Sanders ME, Chakravarthy AB, Shyr Y, et al. Identification of human triple-negative breast cancer subtypes and preclinical models for selection of targeted therapies. J Clin Invest. 2011;121:2750–67. 21633166 10.1172/JCI45014 PMC3127435

[10] Burstein MD, Tsimelzon A, Poage GM, Covington KR, Contreras A, Fuqua SA, et al. Comprehensive genomic analysis identifies novel subtypes and targets of triple-negative breast cancer. Clin Cancer Res. 2015;21:1688–98. 25208879 10.1158/1078-0432.CCR-14-0432 PMC4362882

[11] Bareche Y, Venet D, Ignatiadis M, Aftimos P, Piccart M, Rothe F, et al. Unravelling triple-negative breast cancer molecular heterogeneity using an integrative multiomic analysis. Ann Oncol. 2018;29:895–902. 29365031 10.1093/annonc/mdy024 PMC5913636

[12] Lehmann BD, Jovanović B, Chen X, Estrada MV, Johnson KN, Shyr Y, et al. Refinement of triple-negative breast cancer molecular subtypes: implications for neoadjuvant chemotherapy selection. PLOS ONE. 2016;11:e0157368. 27310713 10.1371/journal.pone.0157368 PMC4911051

[13] Liu YR, Jiang YZ, Xu XE, Yu KD, Jin X, Hu X, et al. Comprehensive transcriptome analysis identifies novel molecular subtypes and subtype-specific RNAs of triple-negative breast cancer. Breast Cancer Res. 2016;18:33. 26975198 10.1186/s13058-016-0690-8 PMC4791797

[14] Gong TQ, Jiang YZ, Shao C, Peng WT, Liu MW, Li DQ, et al. Proteome-centric cross-omics characterization and integrated network analyses of triple-negative breast cancer. Cell Rep. 2022;38:110460. 35235781 10.1016/j.celrep.2022.110460

[15] Jiang YZ, Ma D, Suo C, Shi J, Xue M, Hu X, et al. Genomic and transcriptomic landscape of triple-negative breast cancers: subtypes and treatment strategies. Cancer Cell. 2019;35:428–40.e5. 30853353 10.1016/j.ccell.2019.02.001

[16] Lehmann BD, Colaprico A, Silva TC, Chen J, An H, Ban Y, et al. Multi-omics analysis identifies therapeutic vulnerabilities in triple-negative breast cancer subtypes. Nat Commun. 2021;12:6276. 34725325 10.1038/s41467-021-26502-6 PMC8560912

[17] Masuda H, Harano K, Miura S, Wang Y, Hirota Y, Harada O, et al. Changes in triple-negative breast cancer molecular subtypes in patients without pathologic complete response after neoadjuvant systemic chemotherapy. JCO Precis Oncol. 2022;6:e2000368. 35294223 10.1200/PO.20.00368 PMC8939918

[18] Zanella ER, Grassi E, Trusolino L. Towards precision oncology with patient-derived xenografts. Nat Rev Clin Oncol. 2022;19:719–32. 36151307 10.1038/s41571-022-00682-6

[19] Guillen KP, Fujita M, Butterfield AJ, Scherer SD, Bailey MH, Chu Z, et al. A human breast cancer-derived xenograft and organoid platform for drug discovery and precision oncology. Nat cancer. 2022;3:232–50. 35221336 10.1038/s43018-022-00337-6 PMC8882468

[20] Powell RT, Redwood A, Liu X, Guo L, Cai S, Zhou X, et al. Pharmacologic profiling of patient-derived xenograft models of primary treatment-naïve triple-negative breast cancer. Sci Rep. 2020;10:17899. 33087803 10.1038/s41598-020-74882-4 PMC7578025

[21] Gómez-Miragaya J, Palafox M, Paré L, Yoldi G, Ferrer I, Vila S, et al. Resistance to Taxanes in triple-negative breast cancer associates with the dynamics of a CD49f+ tumor-initiating population. Stem Cell Rep. 2017;8:1392–407. 10.1016/j.stemcr.2017.03.026 PMC5425727 28457887

[22] Bergamaschi A, Hjortland GO, Triulzi T, Sorlie T, Johnsen H, Ree AH, et al. Molecular profiling and characterization of luminal-like and basal-like in vivo breast cancer xenograft models. Mol Oncol. 2009;3:469–82. 19713161 10.1016/j.molonc.2009.07.003 PMC5527532

[23] Lindholm EM, Kristian A, Nalwoga H, Krüger K, Nygård S, Akslen LA, et al. Effect of antiangiogenic therapy on tumor growth, vasculature and kinase activity in basal- and luminal-like breast cancer xenografts. Mol Oncol. 2012;6:418–27. 22521242 10.1016/j.molonc.2012.03.006 PMC5528356

[24] Kristian A, Holtedahl JE, Torheim T, Futsaether C, Hernes E, Engebraaten O, et al. Dynamic 2-Deoxy-2-[18F]Fluoro-D-Glucose Positron Emission Tomography for Chemotherapy Response Monitoring of Breast Cancer Xenografts. Mol Imaging Biol. 2017;19:271–9. 27541026 10.1007/s11307-016-0998-x

[25] Pettersen S, Øy GF, Egeland EV, Juell S, Engebråten O, Mælandsmo GM, et al. Breast cancer patient-derived explant cultures recapitulate in vivo drug responses. Front Oncol. 2023;13:1040665. 36910663 10.3389/fonc.2023.1040665 PMC9992973

[26] Szklarczyk D, Kirsch R, Koutrouli M, Nastou K, Mehryary F, Hachilif R, et al. The STRING database in 2023: protein-protein association networks and functional enrichment analyses for any sequenced genome of interest. Nucleic Acids Res. 2023;51:D638–d46. 36370105 10.1093/nar/gkac1000 PMC9825434

[27] Shannon P, Markiel A, Ozier O, Baliga NS, Wang JT, Ramage D, et al. Cytoscape: a software environment for integrated models of biomolecular interaction networks. Genome Res. 2003;13:2498–504. 14597658 10.1101/gr.1239303 PMC403769

[28] Siwak DR, Li J, Akbani R, Liang H, Lu Y. Analytical Platforms 3: Processing Samples via the RPPA Pipeline to Generate Large-Scale Data for Clinical Studies. In: Yamada T, Nishizuka SS, Mills GB, Liotta LA, editors. Reverse Phase Protein Arrays: From Technical and Analytical Fundamentals to Applications. Singapore: Springer Singapore; 2019. p. 113-47. 10.1007/978-981-32-9755-5_7 31820386

[29] Shehwana H, Kumar SV, Melott JM, Rohrdanz MA, Wakefield C, Ju Z, et al. RPPA SPACE: an R package for normalization and quantitation of Reverse-Phase Protein Array data. Bioinforma (Oxf, Engl). 2022;38:5131–3. 10.1093/bioinformatics/btac665 PMC9665860 36205581

[30] Akbani R, Ng PK, Werner HM, Shahmoradgoli M, Zhang F, Ju Z, et al. A pan-cancer proteomic perspective on The Cancer Genome Atlas. Nat Commun. 2014;5:3887. 24871328 10.1038/ncomms4887 PMC4109726

[31] Silwal-Pandit L, Nord S, von der Lippe Gythfeldt H, Moller EK, Fleischer T, Rodland E, et al. The longitudinal transcriptional response to neoadjuvant chemotherapy with and without bevacizumab in breast cancer. Clin Cancer Res. 2017;23:4662–70. 28487444 10.1158/1078-0432.CCR-17-0160

[32] Haugen MH, Lingjærde OC, Hedenfalk I, Garred Ø, Borgen E, Loman N, et al. Protein Signature Predicts Response to Neoadjuvant Treatment With Chemotherapy and Bevacizumab in HER2-Negative Breast Cancers. JCO Precision Oncology. 2021:286-306. 10.1200/PO.20.00086 PMC8140811 34036235

[33] Robinson DR, Wu YM, Lonigro RJ, Vats P, Cobain E, Everett J, et al. Integrative clinical genomics of metastatic cancer. Nature. 2017;548:297–303. 28783718 10.1038/nature23306 PMC5995337

[34] Aran D, Hu Z, Butte AJ. xCell: digitally portraying the tissue cellular heterogeneity landscape. Genome Biol. 2017;18:220. 29141660 10.1186/s13059-017-1349-1 PMC5688663

[35] Horwitz SB, Cohen D, Rao S, Ringel I, Shen HJ, Yang CP. Taxol: mechanisms of action and resistance. Journal of the National Cancer Institute Monographs. 1993:55-61. 7912530

[36] Aguilera P, López-Contreras AJ. ATRX, a guardian of chromatin. Trends Genet : TIG. 2023;39:505–19. 36894374 10.1016/j.tig.2023.02.009

[37] Roskoski R Jr. Src protein-tyrosine kinase structure, mechanism, and small molecule inhibitors. Pharmacol Res. 2015;94:9–25. 25662515 10.1016/j.phrs.2015.01.003

[38] Luo J, Zou H, Guo Y, Tong T, Ye L, Zhu C, et al. SRC kinase-mediated signaling pathways and targeted therapies in breast cancer. Breast Cancer Res. 2022;24:99. 36581908 10.1186/s13058-022-01596-y PMC9798727

[39] Xu X, Kumari R, Zhou J, Chen J, Mao B, Wang J, et al. A living biobank of matched pairs of patient-derived xenografts and organoids for cancer pharmacology. PLoS One. 2023;18:e0279821. 36602988 10.1371/journal.pone.0279821 PMC9815646

[40] Poh AR, Ernst M. Functional roles of SRC signaling in pancreatic cancer: recent insights provide novel therapeutic opportunities. Oncogene. 2023;42:1786–801. 37120696 10.1038/s41388-023-02701-x PMC10238273

[41] Engle K, Kumar G. Cancer multidrug-resistance reversal by ABCB1 inhibition: a recent update. Eur J medicinal Chem. 2022;239:114542. 10.1016/j.ejmech.2022.114542 35751979

[42] Kohale IN, Yu J, Zhuang Y, Fan X, Reddy RJ, Sinnwell J, et al. Identification of Src Family Kinases as Potential Therapeutic Targets for Chemotherapy-Resistant Triple Negative Breast Cancer. Cancers. 2022;14. 10.3390/cancers14174220 PMC9454481 36077757

[43] Zhang XH, Wang Q, Gerald W, Hudis CA, Norton L, Smid M, et al. Latent bone metastasis in breast cancer tied to Src-dependent survival signals. Cancer Cell. 2009;16:67–78. 19573813 10.1016/j.ccr.2009.05.017 PMC2749247

[44] Gucalp A, Sparano JA, Caravelli J, Santamauro J, Patil S, Abbruzzi A, et al. Phase II trial of saracatinib (AZD0530), an oral SRC-inhibitor for the treatment of patients with hormone receptor-negative metastatic breast cancer. Clin breast cancer. 2011;11:306–11. 21729667 10.1016/j.clbc.2011.03.021 PMC3222913

[45] Schott AF, Barlow WE, Van Poznak CH, Hayes DF, Moinpour CM, Lew DL, et al. Phase II studies of two different schedules of dasatinib in bone metastasis predominant metastatic breast cancer: SWOG S0622. Breast Cancer Res Treat. 2016;159:87–95. 27475087 10.1007/s10549-016-3911-z PMC5021222

[46] Somlo G, Atzori F, Strauss LC, Geese WJ, Specht JM, Gradishar WJ, et al. Dasatinib plus capecitabine for advanced breast cancer: safety and efficacy in phase I study CA180004. Clin Cancer Res. 2013;19:1884–93. 23403636 10.1158/1078-0432.CCR-12-0652

[47] Morris PG, Rota S, Cadoo K, Zamora S, Patil S, D’Andrea G, et al. Phase II study of paclitaxel and dasatinib in metastatic breast cancer. Clin breast cancer. 2018;18:387–94. 29680193 10.1016/j.clbc.2018.03.010 PMC6682312

[48] Moulder S, Yan K, Huang F, Hess KR, Liedtke C, Lin F, et al. Development of candidate genomic markers to select breast cancer patients for dasatinib therapy. Mol Cancer Ther. 2010;9:1120–7. 20423993 10.1158/1535-7163.MCT-09-1117

[49] Ortiz MA, Mikhailova T, Li X, Porter BA, Bah A, Kotula L. SRC family kinases, adaptor proteins and the actin cytoskeleton in epithelial-to-mesenchymal transition. Cell Commun Signal : CCS. 2021;19:67. 34193161 10.1186/s12964-021-00750-x PMC8247114

[50] Temps C, Lietha D, Webb ER, Li XF, Dawson JC, Muir M, et al. A conformation selective mode of inhibiting SRC improves drug efficacy and tolerability. Cancer Res. 2021;81:5438–50. 34417202 10.1158/0008-5472.CAN-21-0613 PMC7611940

[51] Kim LC, Song L, Haura EB. Src kinases as therapeutic targets for cancer. Nat Rev Clin Oncol. 2009;6:587–95. 19787002 10.1038/nrclinonc.2009.129

[52] Chin YR, Yoshida T, Marusyk A, Beck AH, Polyak K, Toker A. Targeting Akt3 signaling in triple-negative breast cancer. Cancer Res. 2014;74:964–73. 24335962 10.1158/0008-5472.CAN-13-2175 PMC3946502

