# Supplementary material for: The SRC-family serves as a therapeutic target in triple negative breast cancer with acquired resistance to chemotherapy
Source: Br J Cancer. 2024 Oct 10;131(10):1656–67. doi: 10.1038/s41416-024-02875-5 (PMC11554838; doi:10.1038/s41416-024-02875-5)
Supplement: Supplementary file 1 — Supplementary methods [file 41416_2024_2875_MOESM1_ESM.docx]

***Supplementary methods***

**DNA variant calling and annotation**

Short sequencing reads from whole-exome sequencing were preprocessed and aligned against the human reference genome (grch38 assembly) using the *Sarek nf-core workflow* (v3.1.1) [1, 2]. Somatic single nucleotide variants and short insertions/deletions were identified through MuTect2 in a tumor-only mode, here also using the Sarek workflow [3]. Variants were next functionally annotated through the Variant Effect Predictor (*VEP* v109), using GENCODE (v43) as the gene and transcript reference [4, 5]. Various other annotations to inform upon functional variants were also appended, including information on mutational hotspots in cancer, loss-of-function estimation from the LOFTEE algorithm, as well as variant effect predictions (i.e. tolerated vs. damaging) from *dbNSFP* (v4.2) and *dbscSNV* (v1.1) [6-9]. The potential role of genes as proto-oncogenes/tumor suppressors were retrieved from the curated Cancer Gene Census resource (v97) and the literature-derived CancerMine database (v50) [10, 11]. The potential oncogenic effect of variants was evaluated by implementing recently proposed recommendations for classification of oncogenicity of somatic variants [12].

Tumor mutational burden (TMB) per sample was calculated as the number of coding somatic mutations (including silent) divided by the approximate coding size of the human exome (34 Mb).

**RNA sequencing**

*RNA sequencing data processing*

Samples were cropped with *Trimmomatic* (v0.38, [13]) to the longest common read length, 75 bp, to reduce read mapping bias. Sequence reads were aligned to the human genome reference (grch38) with *STAR* (v2.7.8a, [14]), using GENCODE (v41) as the transcript reference. Default parameters were used, and NM distance was included in the output. To separate mouse from human sequence reads, sequence reads were aligned to the mouse genome reference (mm10) separately, with transcript reference GENCODE (vM25) and the same setting as for the human genome. Aligned BAMs were used to exclude reads which either strictly mapped to the mouse genome or showed a better mapping than the human read alignment with the R package *XenofFilteR* (v1.6) [15] in R. Finally, gene counts and gene lengths were retrieved from the filtered BAM with *featureCounts* included in the *Subread* package (v2.0.1, [16]), using GENCODE (v41) as the transcript reference. *FastQC* (v0.11.9) and *RSeQC* (v2.6.4) were used to assess quality of samples pre- and post-alignment. RNAseq data processing was performed in the Galaxy environment (https://usegalaxy.no/ [17]), unless otherwise specified.

*RNA sequencing analyses*

Read counts were processed with the R package *DESeq2* (v1.40.2). Differentially expressed genes were determined with the function *DESeq*, including batch in the design to account for sequencing batch effects. Default settings were used, and *lfcShrink* with the method *ashr* was applied to shrink log fold change (LFC) values towards 0. For principal component analysis (PCA) plot and Heatmaps, variance stabilized transformated (vst) data were generated by estimating size factors and dispersion with functions *estimateSizeFactors* and *estimateDispersions*, followed by *varianceStabilizingTransformation*. Batch effects were then accounted for with the function *removeBatchEffect* from the *limma* (v3.56.2) package.

For evaluating gene expression across samples transcripts per million (*tpm*) were calculated with the formula, using read counts and corresponding gene length:

$${tpm}_{i}=\frac{\left( \frac{{Counts}_{i}}{{Gene Length}_{i}} \right)}{\sum_{j} \left( \frac{{Counts}_{j}}{{Gene Length}_{j}} \right)}\times{10}^{6}$$

*Gene set enrichment analyses*

Gene set enrichment analyses (GSEA) were done with the R package *fgsea* (v1.26.0) and function *fgsea* with settings *minSize=15* and *maxSize=500*. The respective gene sets were imported from the R package *msigdbr* (v7.5.1).

*Heatmap*

Heatmap were generated with vst-transformed counts using the R packages *pheatmap* (v1.0.12) and *ComplexHeatmap* (v2.16.0). Samples were clustered with distance *correlation* with method *average*. For annotation of cluster function the R package *GeneOverlap* (v1.36.0) was used to calculate overlap between gene clusters and *Hallmark* and *KEGG* gene sets. Cluster annotation was then manually curated, to summarize the most significant functional characteristic.

*TNBC subtyping*

TNBC subtyping was performed by uploading *tpm* values to the web-based *TNBCsubtype* (http://cbc.mc.vanderbilt.edu/tnbc/) to classify parental and chemo-resistant MAS98.12 into the four TNBC subtypes defined by Lehmann *et al.* [18] by centroid correlation. For accurate determination, the PDX tumors were subtyped together with RNAseq data from 72 TNBC primary tumors (data unpublished).

**Immunohistochemistry (IHC)**

IHC was performed on 3 µm sections of formalin-fixed paraffin-embedded PDX tissue. For EPCAM, CK19, E-CADHERIN and VIMENTIN, deparaffinization, rehydration and target retrieval were performed on Dako PT-link system in EnVision™ Flex target retrieval solution (Dako, Glostrup, Denmark). After treatment with Dako EnVision Peroxidase Block, the sections were stained with the primary antibodies as specified in Supplementary Table S3. Subsequently, the sections were incubated with Dako EnVision™ +System-HRP and further developed with DAB Chromogen (Dako, Santa Clara, CA) and counterstained with hematoxylin.

IHC for Ki67, MDR1 and SRC pY416 was performed manually. Deparaffinization was performed using Neo-clear (VWR, Radnor, PA). For epitope retrieval, the slides were immersed in 10 mM Tris/1 mM EDTA buffer (pH 9) at 100 ^°^C for 20 min. After treatment with H_2_O_2_ solution provided with HRP/DAB detection kit (ab236469, Abcam, Cambridge, UK), the blocking was performed using protein block provided with the kit before staining with the primary antibodies (specified in Supplementary Table S3) for 1h at room temperature. For detection, DAB chromogen solution provided with the kit was used, and counterstaining was performed using hematoxylin (Sigma-Aldrich). The sections were digitalized using Olympus VS200 Slide Scanner (Tokyo, Japan), using a 20X objective. Images were analyzed using QuPath-0.5.0 software (open source).

**Preparation of protein lysates from PDXC**

For PDXC, the cultured tissue was collected by centrifugation and mixed with 20-30 µL of the lysis buffer (as described in Methods section) before being lysed by incubation on ice for 30 min, vortexing every 5-10min. After centrifugation at 14.000rpm for 10 min at 4^o^C, the supernatant was collected, and the protein concentration was determined by using a BCA protein assay kit (Thermo Fisher Scientific).

**Immunofluorescence (IF)**

For IF, PDXCs were fixed in 4% PFA (Chemi-Teknik, Oslo, Norway) for 15 min followed by 20 min permeabilization with 0.5% Triton X-100 (Merck, Darmstadt, Germany) and 1 h blocking in 5% horse serum (Gibco, Grand Island, NY) in IF buffer (phosphate-buffered saline (PBS) with 0.1% bovine serum albumin (BSA) (both Sigma-Aldrich), 0.2% Triton X-100 and 0.05% Tween-20 (Merck). The samples were incubated with primary antibodies, (specified in Supplementary Table S3) in IF buffer overnight at 4°C. After washing in IF buffer, the samples were incubated with secondary antibodies and DAPI (specified in Supplementary Table S3) in IF buffer for 3 h at room temperature. Imaging was performed using Olympus IX81microscope equipped with a 10x objective (Olympus, Tokyo, Japan).

**Live/dead staining**

PDXCs were stained with 1 µM calcein-AM (Sigma-Aldrich) for 30 min at 37°C followed by staining with 350 nM propidium iodide (PI) (Invitrogen, Waltham, MA, USA) for 30 min to distinguish live (green) and dead (red) cells, respectively. The stained cultures were analyzed by Olympus IX81 microscope equipped with a 4x objective and filters 488/527 (for calcein) and 540/590 (for PI; Olympus, Tokyo, Japan). The images covering whole area of the dome were captured and analyzed with Fiji/ImageJ (an open-source software for image processing [19], measuring the calcein-signal area in pixels, which reflects “live” tissue area.

**Clinical cohorts for primary and treated TNBC**

*TCGA*

For TCGA data was downloaded from the GDC database, using the *TCGAbiolinks* (v2.28.4) R package. Samples defined as TNBC in Lehmann *et al* [20] was retrieved for survival analyses, and RNAseq raw counts were imported into *DESeq2* and vst-transformed as described above.

*METABRIC*

METABRIC log2 gene expression intensity data was downloaded from cBioPortal. Data was quantile normalized, and median centered, prior to survival analyses. Clinical data was retrieved for TNBC from Lehmann *et al*. [20].

*GSE123845 dataset*

Data from the study by Park *et al* [21], containing gene expression from patients before and after neoadjuvant therapy (NAT), were downloaded from the Gene Expression Omnibus (GSE123845) as *tpm* values, and log2-transformed. Only samples with available data at both baseline and surgery (post-NAT) were included, giving a total of eight paired TNBC samples.

**References:**

[1] Ewels PA, Peltzer A, Fillinger S, Patel H, Alneberg J, Wilm A, et al. The nf-core framework for community-curated bioinformatics pipelines. Nature Biotechnology. 2020;38:276-8.

[2] Garcia M, Juhos S, Larsson M, Olason PI, Martin M, Eisfeldt J, et al. Sarek: A portable workflow for whole-genome sequencing analysis of germline and somatic variants. F1000Research. 2020;9:63.

[3] David B, Takuto S, Kristian C, Gad G, Chip S, Lee L. Calling Somatic SNVs and Indels with Mutect2. bioRxiv. 2019:861054.

[4] McLaren W, Gil L, Hunt SE, Riat HS, Ritchie GRS, Thormann A, et al. The Ensembl Variant Effect Predictor. Genome biology. 2016;17:122.

[5] Frankish A, Diekhans M, Jungreis I, Lagarde J, Loveland JE, Mudge JM, et al. GENCODE 2021. Nucleic Acids Res. 2021;49:D916-d23.

[6] Chang MT, Bhattarai TS, Schram AM, Bielski CM, Donoghue MTA, Jonsson P, et al. Accelerating Discovery of Functional Mutant Alleles in Cancer. Cancer Discov. 2018;8:174-83.

[7] Karczewski KJ, Francioli LC, Tiao G, Cummings BB, Alföldi J, Wang Q, et al. The mutational constraint spectrum quantified from variation in 141,456 humans. Nature. 2020;581:434-43.

[8] Liu X, Li C, Mou C, Dong Y, Tu Y. dbNSFP v4: a comprehensive database of transcript-specific functional predictions and annotations for human nonsynonymous and splice-site SNVs. Genome Medicine. 2020;12:103.

[9] Jian X, Boerwinkle E, Liu X. In silico prediction of splice-altering single nucleotide variants in the human genome. Nucleic Acids Res. 2014;42:13534-44.

[10] Sondka Z, Bamford S, Cole CG, Ward SA, Dunham I, Forbes SA. The COSMIC Cancer Gene Census: describing genetic dysfunction across all human cancers. Nat Rev Cancer. 2018;18:696-705.

[11] Lever J, Zhao EY, Grewal J, Jones MR, Jones SJM. CancerMine: a literature-mined resource for drivers, oncogenes and tumor suppressors in cancer. Nat Methods. 2019;16:505-7.

[12] Horak P, Griffith M, Danos AM, Pitel BA, Madhavan S, Liu X, et al. Standards for the classification of pathogenicity of somatic variants in cancer (oncogenicity): Joint recommendations of Clinical Genome Resource (ClinGen), Cancer Genomics Consortium (CGC), and Variant Interpretation for Cancer Consortium (VICC). Genetics in medicine : official journal of the American College of Medical Genetics. 2022;24:986-98.

[13] Bolger AM, Lohse M, Usadel B. Trimmomatic: a flexible trimmer for Illumina sequence data. Bioinformatics (Oxford, England). 2014;30:2114-20.

[14] Dobin A, Davis CA, Schlesinger F, Drenkow J, Zaleski C, Jha S, et al. STAR: ultrafast universal RNA-seq aligner. Bioinformatics (Oxford, England). 2013;29:15-21.

[15] Kluin RJC, Kemper K, Kuilman T, de Ruiter JR, Iyer V, Forment JV, et al. XenofilteR: computational deconvolution of mouse and human reads in tumor xenograft sequence data. BMC bioinformatics. 2018;19:366.

[16] Liao Y, Smyth GK, Shi W. The Subread aligner: fast, accurate and scalable read mapping by seed-and-vote. Nucleic Acids Res. 2013;41:e108.

[17] Community TG. The Galaxy platform for accessible, reproducible and collaborative biomedical analyses: 2022 update. Nucleic Acids Research. 2022;50:W345-W51.

[18] Lehmann BD, Jovanović B, Chen X, Estrada MV, Johnson KN, Shyr Y, et al. Refinement of Triple-Negative Breast Cancer Molecular Subtypes: Implications for Neoadjuvant Chemotherapy Selection. PLOS ONE. 2016;11:e0157368.

[19] Schneider CA, Rasband WS, Eliceiri KW. NIH Image to ImageJ: 25 years of image analysis. Nature Methods. 2012;9:671-5.

[20] Lehmann BD, Colaprico A, Silva TC, Chen J, An H, Ban Y, et al. Multi-omics analysis identifies therapeutic vulnerabilities in triple-negative breast cancer subtypes. Nat Commun. 2021;12:6276.

[21] Park YH, Lal S, Lee JE, Choi YL, Wen J, Ram S, et al. Chemotherapy induces dynamic immune responses in breast cancers that impact treatment outcome. Nat Commun. 2020;11:6175.
