# Supplementary material for: The SRC-family serves as a therapeutic target in triple negative breast cancer with acquired resistance to chemotherapy
Source: Br J Cancer. 2024 Oct 10;131(10):1656–67. doi: 10.1038/s41416-024-02875-5 (PMC11554838; doi:10.1038/s41416-024-02875-5)

# Supplementary Fig. S1

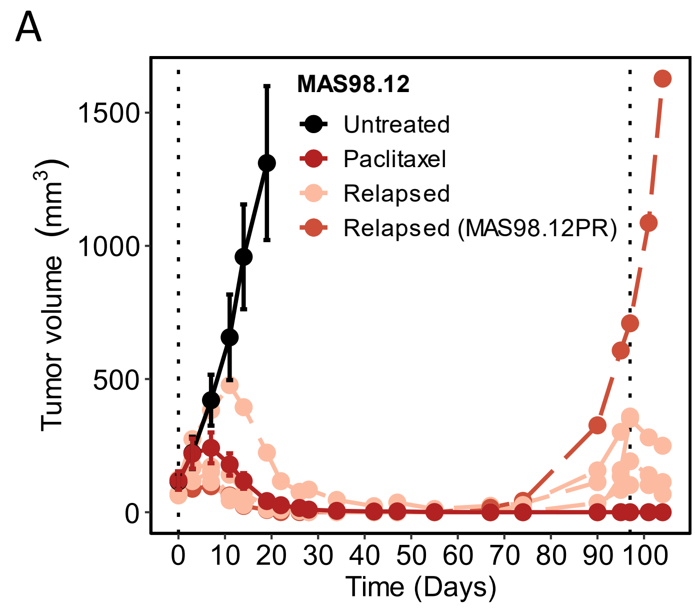


**Supplementary Figure S1. Chemosensitivity of MAS98.12 and MAS98.12PR PDX.** Absolute tumor volume after the treatment specified in Fig. 1. The corresponding relative tumor volumes are shown in Fig. 1A-D.

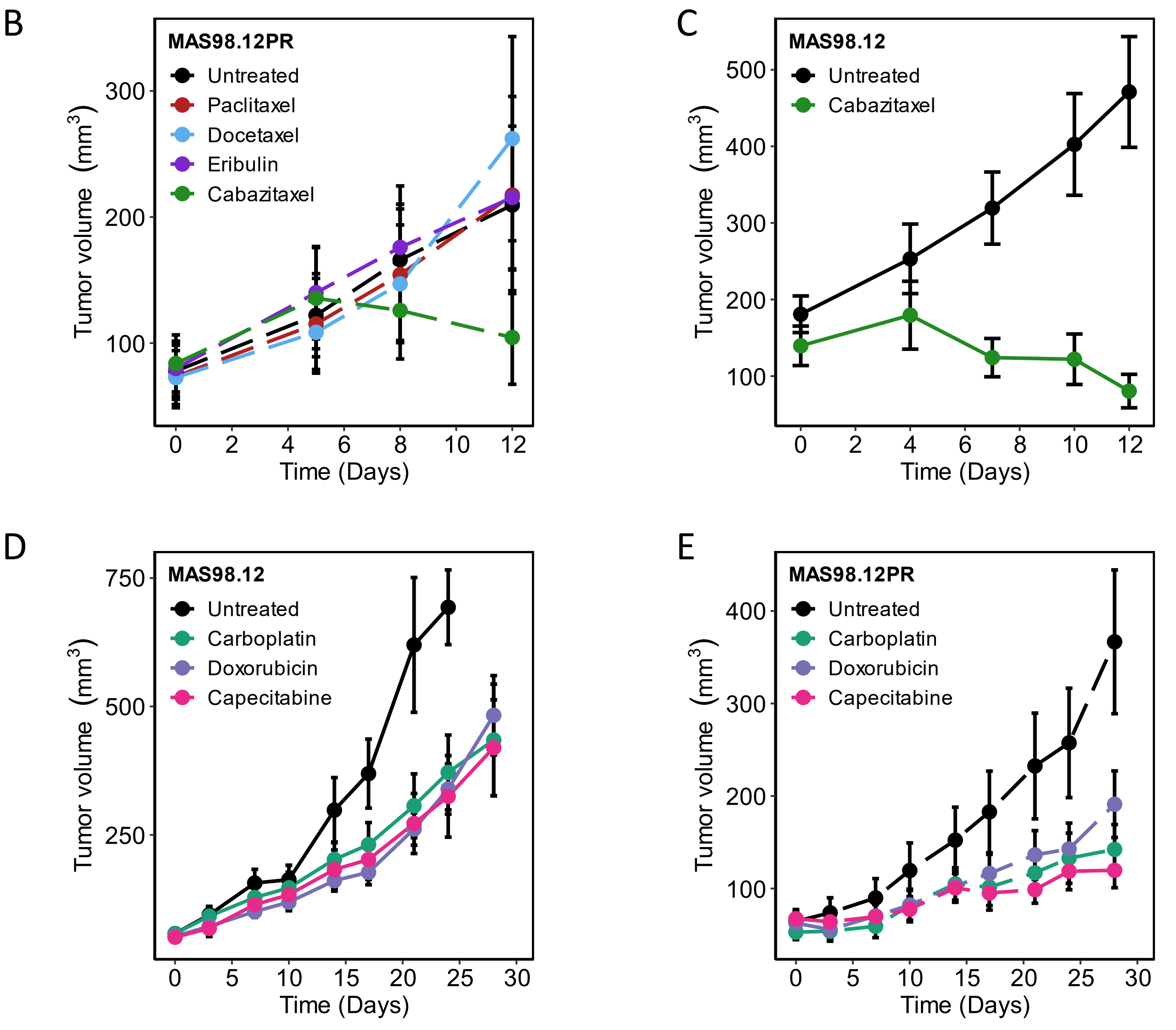

# Supplementary Fig. S2

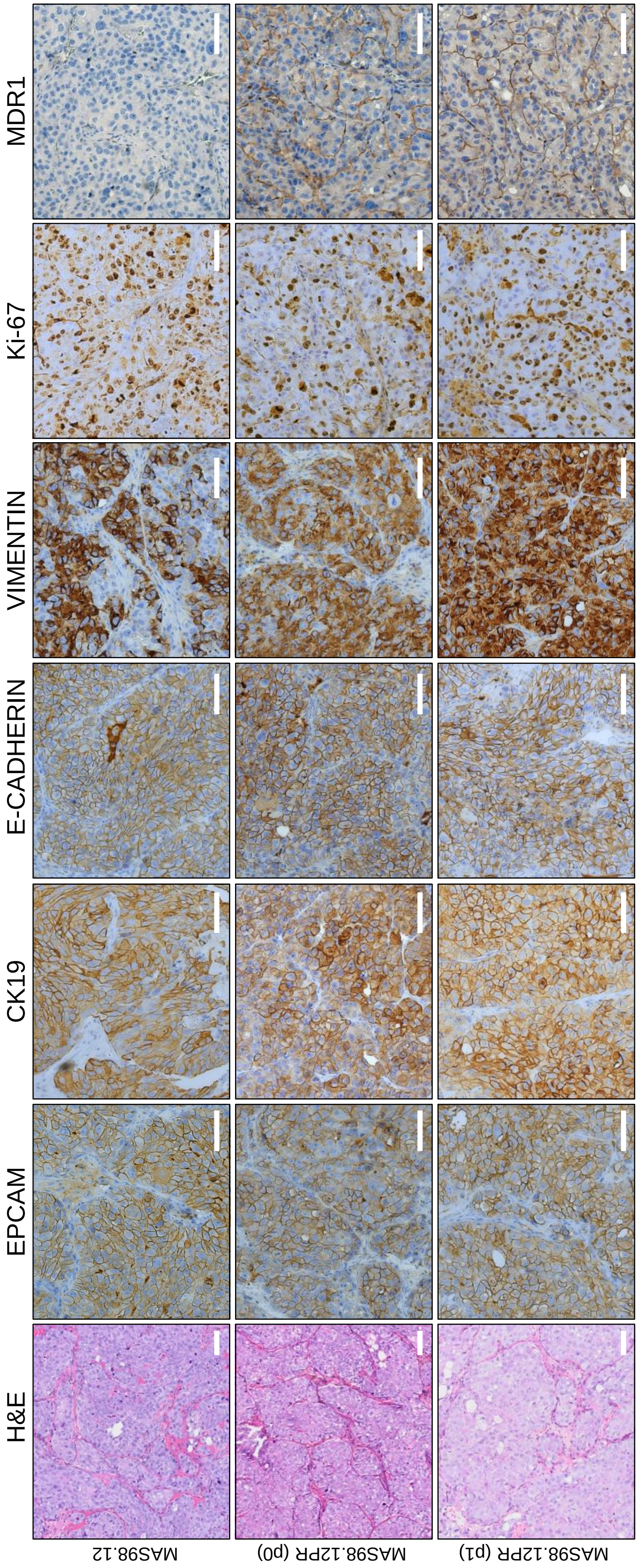


**Supplementary Figure S2. Histopathological features of MAS98.12 and MAS98.12PR** at origin (p0), and after one passage (p1). Scale bar: 100 µm.

# Supplementary Fig. S3

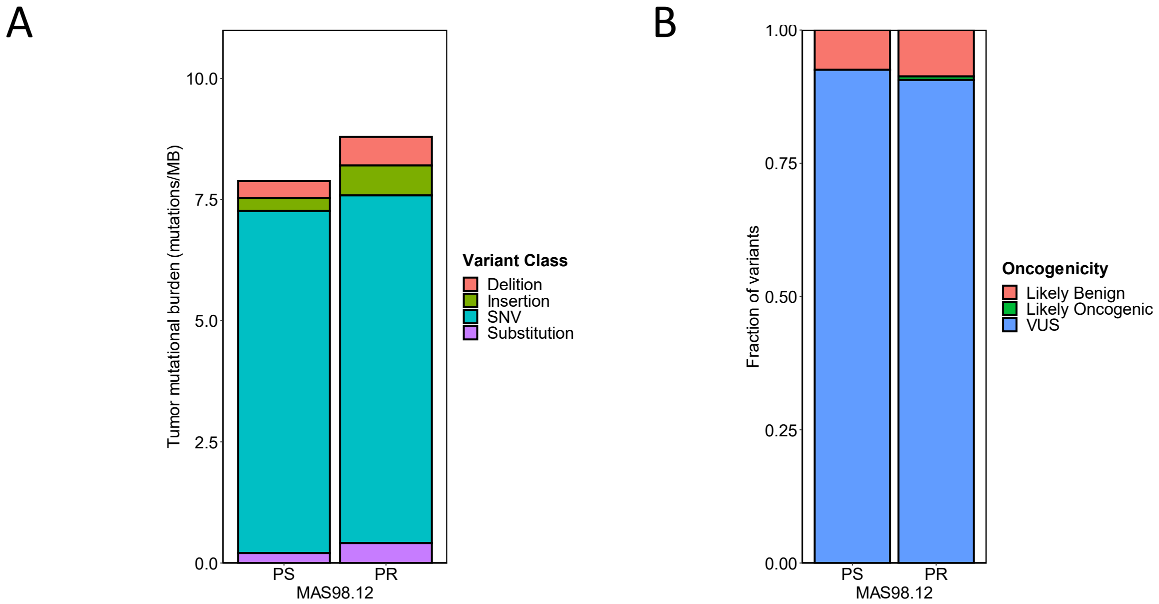


**Supplementary Figure S3. Mutational landscape of MAS98.12 and MAS98.12PR.** A) Tumor mutational burden in MAS98.12 and MAS98.12PR with frequency of distinct classes of the variants highlighted. B) Fraction of variants with distinct predicted oncogenicity *i.e.* likely benign, likely oncogenic or uncertain significance (VUS) in MAS98.12 and MAS98.12PR.

# Supplementary Fig. S4

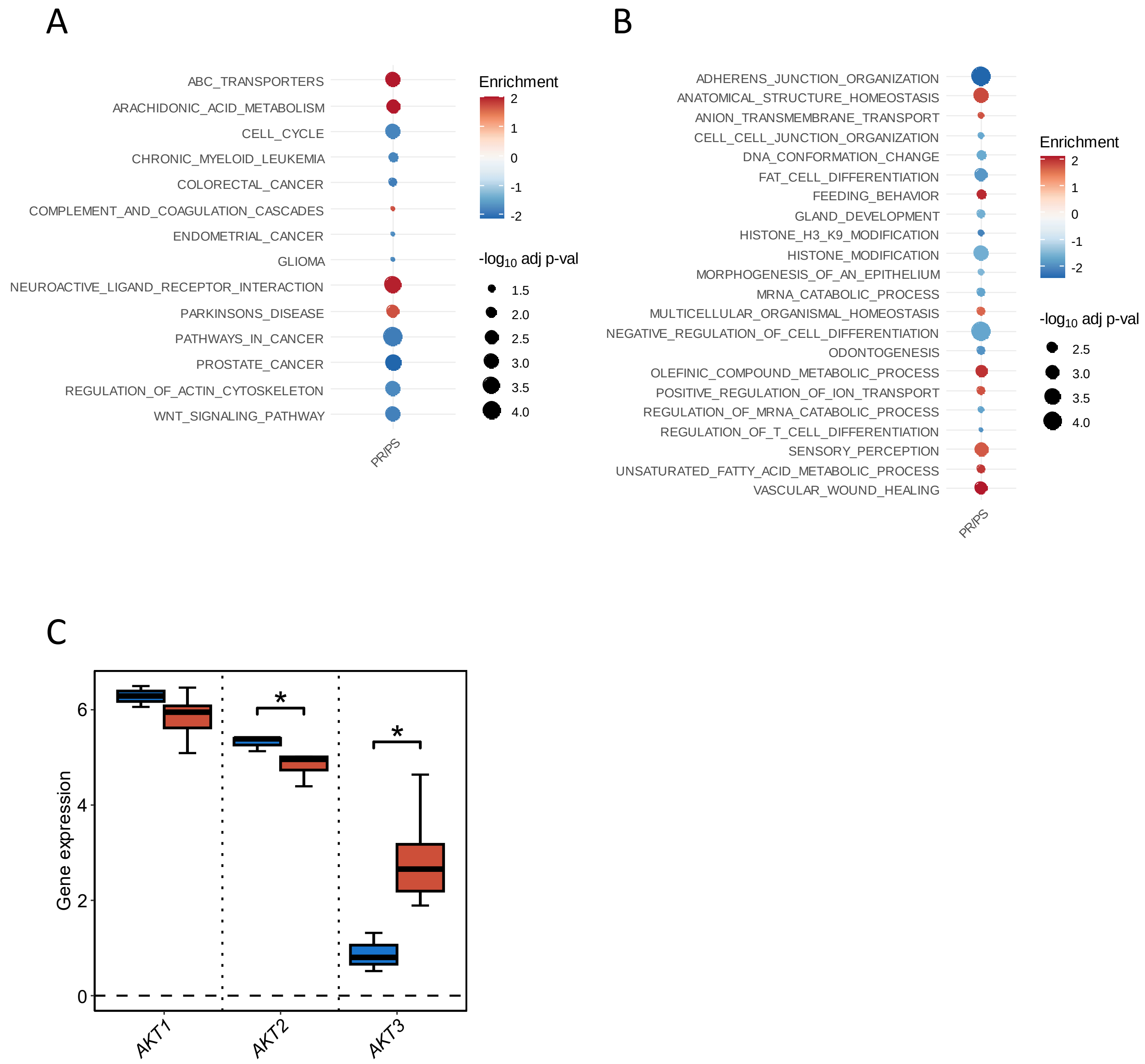


**Supplementary Figure S4. Transcriptional changes in MAS98.12PR compared to MAS98.12.** GSEA using gene sets in KEGG (A) and GO Biological Pathway databases (B). Pathways with p.adj < 0.05 and p.adj < 0.01, respectively, were included. C) Gene expression of distinct isoforms of *AKT* as measured by log2(*tpm*+1).

Supplementary Fig. S5

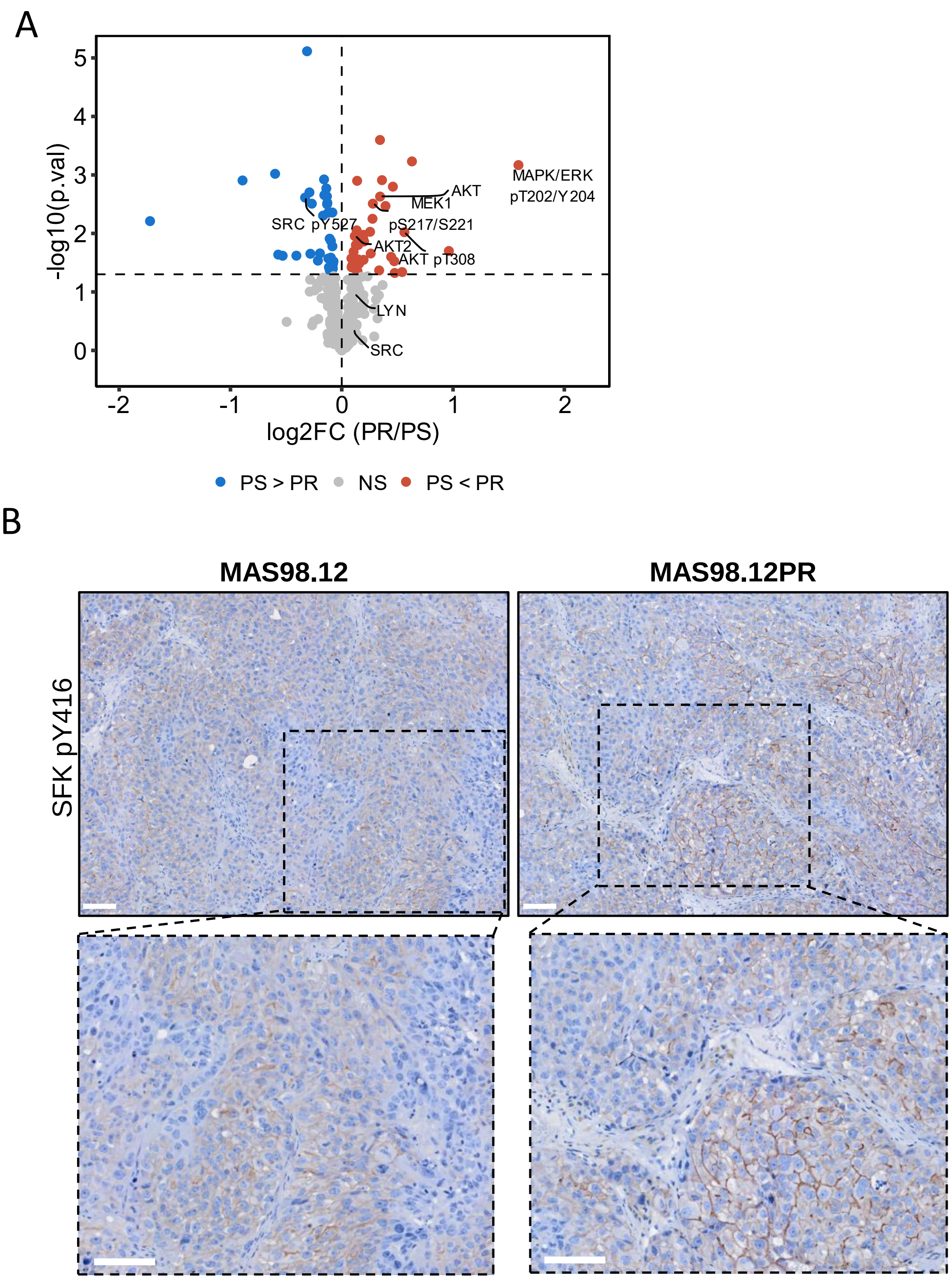


**Supplementary Figure S5. Changes in signaling pathways in MAS98.12PR compared to MAS98.12.** A) Volcano plot of changes in (phospho)proteins in the RPPA data. Dotted lines indicate p-value < 0.05, with red dots indicating higher expression in paclitaxel-resistant , and blue dots indicating higher in paclitaxel-sensitive tumors, proteins associated with SFK-, MAPK/ERK- and AKT-signaling are highlighted. B) IHC of SFK pY416 in MAS98.12 and MAS98.12PR. Scale bar: 100 µm.

Supplementary Fig. S6

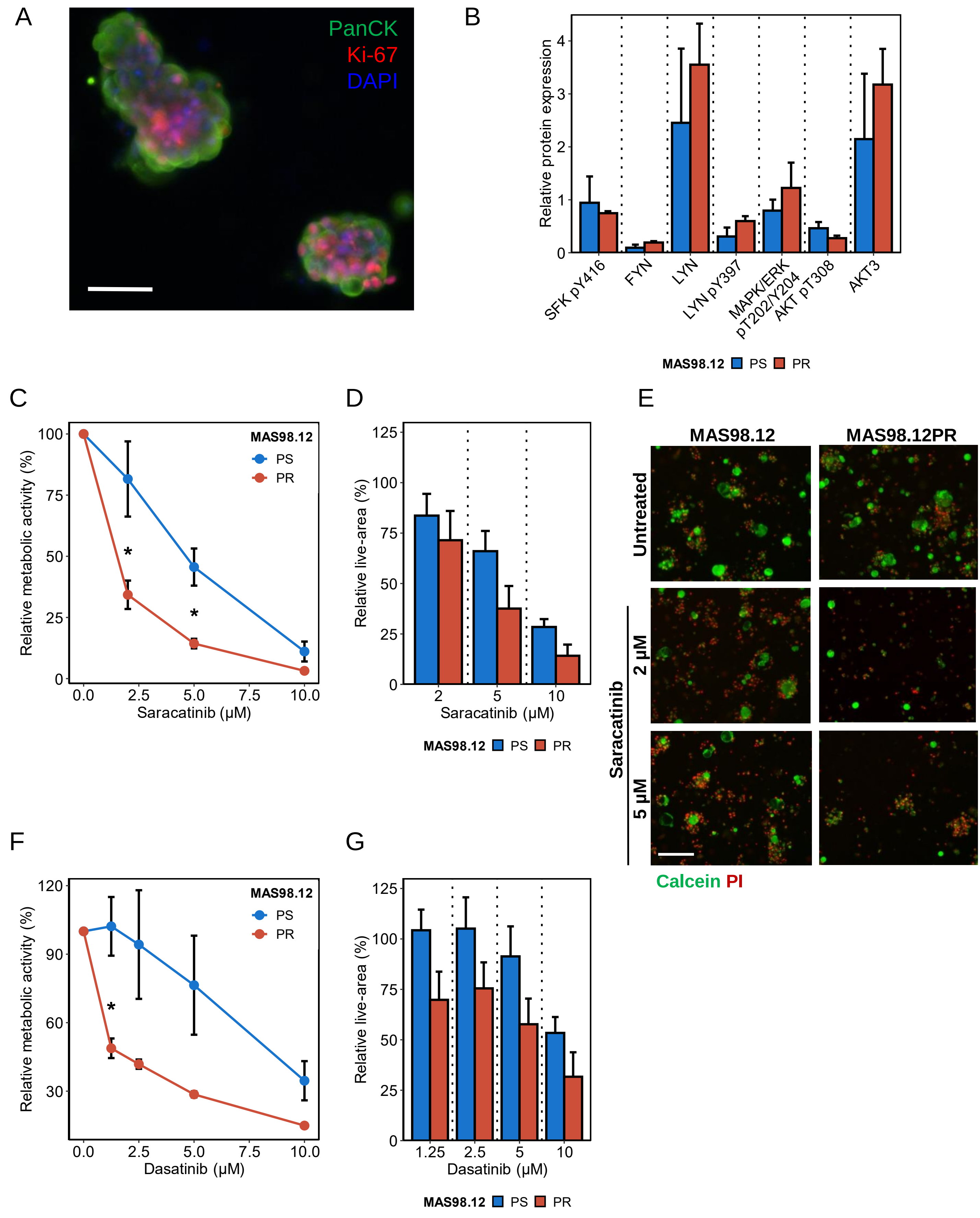

# Supplementary Fig. S6 (continued)

H

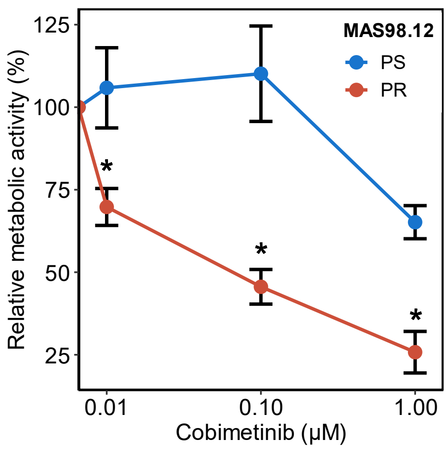


I

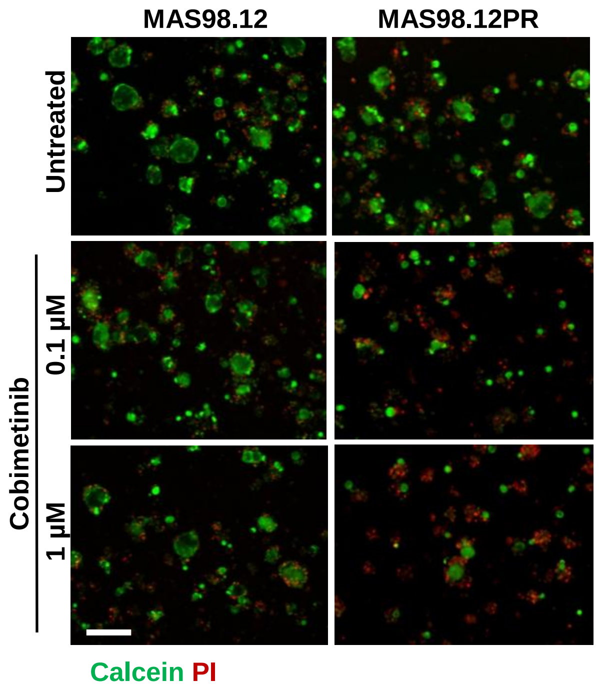


J

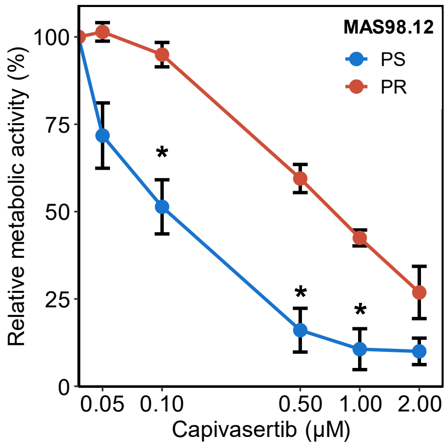


**Supplementary Figure S6. Tumor tissue sensitivity to targeted treatment *ex vivo*.** A) MAS98.12PR tissue cultured as PDXC; IHC for epithelial marker panCK (green) and proliferation marker Ki-67 (red); DAPI (blue) indicates the nucleus; scale bar, 100 μm. B) Relative expression (normalized to the respective loading controls) of proteins from the SFK-, MAPK/ERK- and AKT-pathways in PDXCs from MAS98.12 and MAS98.12PR. C-G) PDXCs from MAS98.12 and MAS98.12PR were treated with different concentrations of saracatinib (C-E) or dasatinib (F-G) for 6 days and the effect was evaluated by measuring tissue metabolic activity (C, F) or live-area (D-G). Data was normalized to the respective untreated controls; mean ± SEM (n= 3-4). E) Representative images indicating live-tissue identified by calcein staining (green) and dead-tissue identified by PI staining (red) in untreated and saracatinib-treated PDXCs. Scale bar: 200μm. (H-I) PDXCs were treated with different concentrations of cobimetinib for 6 days, and the effect was evaluated by measuring tissue metabolic activity and normalizing to the respective untreated controls (H); mean ± SEM (n =6). I) Representative images indicating live-tissue identified by calcein staining (green) and dead-tissue identified by PI staining (red) in untreated and cobimetinib -treated PDXCs. Scale bar: 200 μm. F) PDXCs were treated with different concentrations of capivasertib for 6 days, and the effect was evaluated by measuring tissue metabolic activity and normalizing to the respective untreated controls; mean ± SEM (n =3).

Supplementary Fig. S7

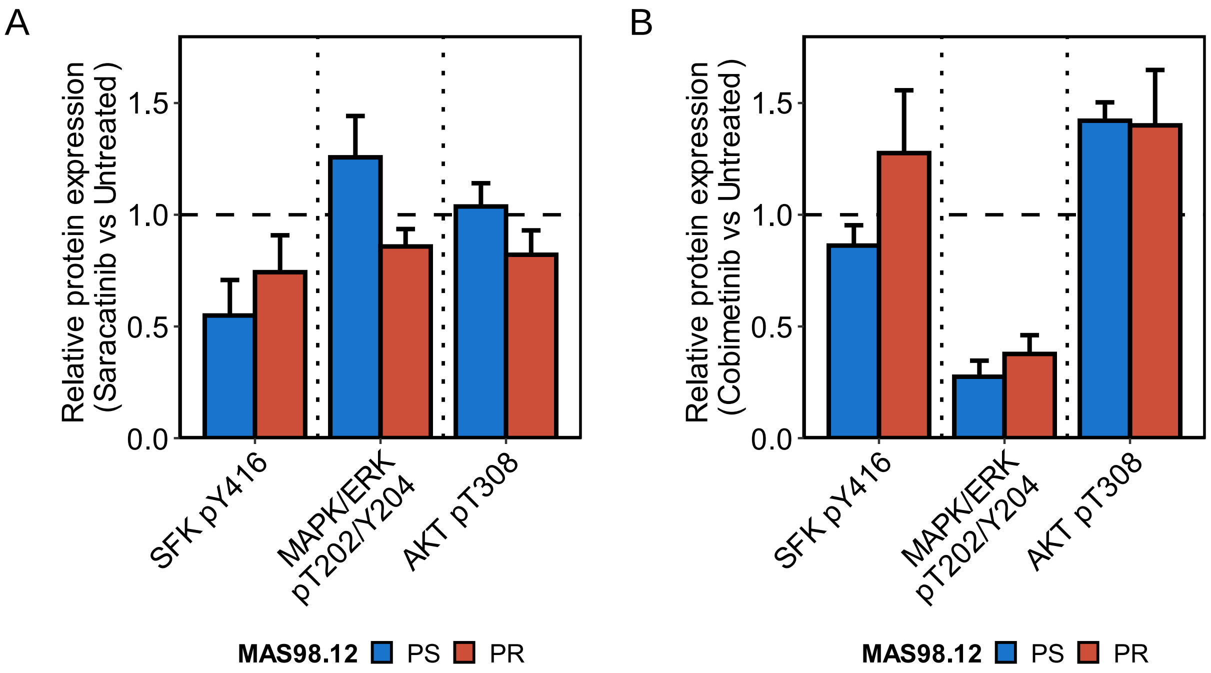


**Supplementary Figure S7. Effect of saracatinib and cobimetinib on phospho-SFK, -ERK and -AKT in PDXC.** Relative level of the indicated proteins in PDXCs exposed to either 2 μM saracatinib (A) or 0.1 μM cobimetinib (B) for 3 hrs before protein lysates were analyzed by SWI. The protein levels in the treated samples were normalized to the levels in the respective untreated controls set to 1 (indicated by dotted lines); mean ± SEM (n=5).

Supplementary Fig. S8

A

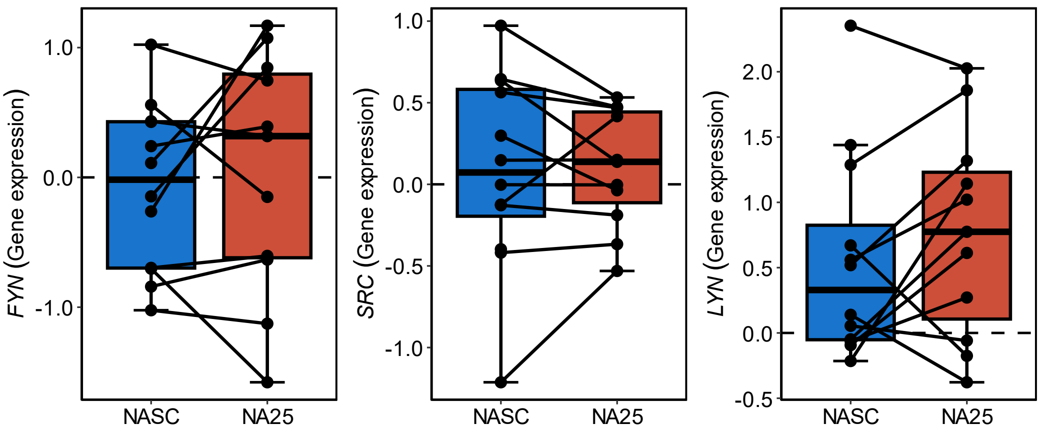


B

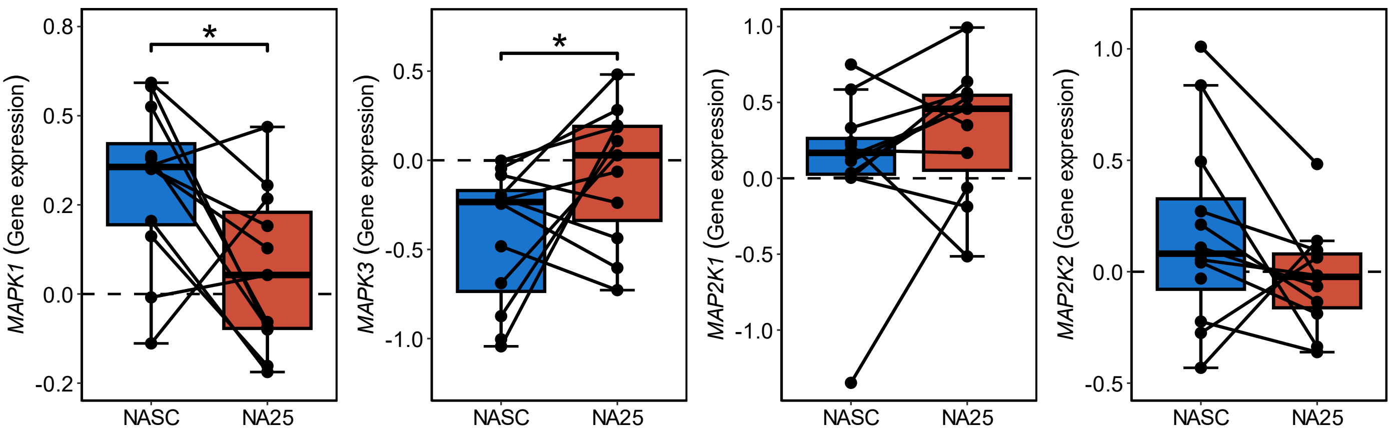

Supplementary Fig. S8 (continued)

C

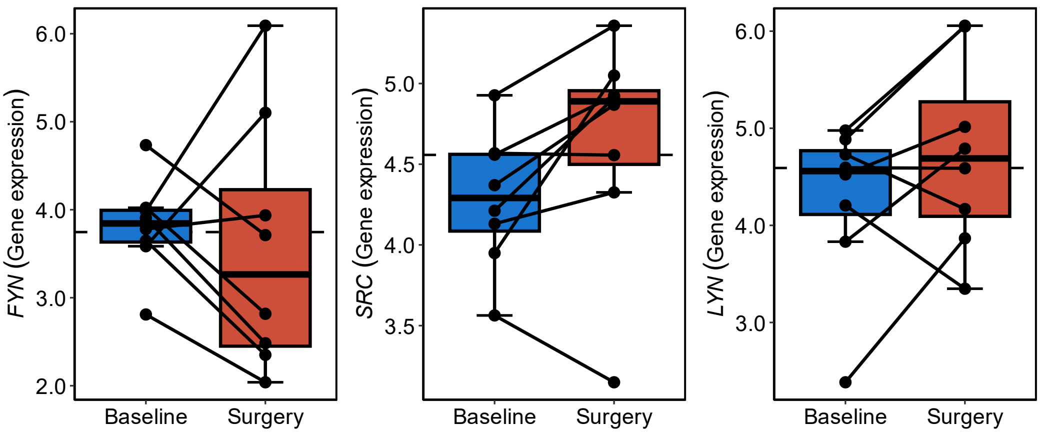


D

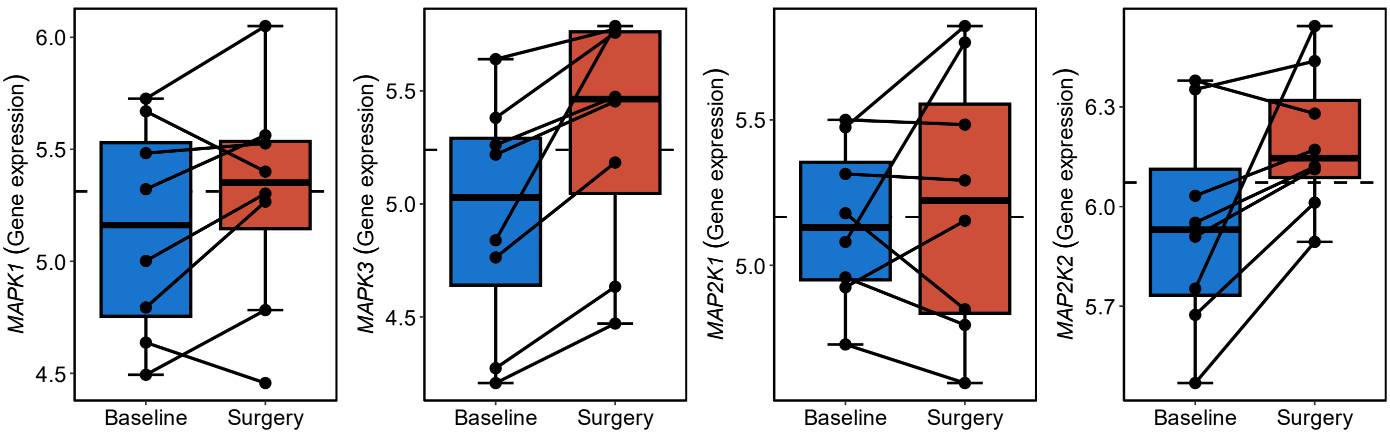


**Supplementary Figure S8. Expression of SFK- and MAPK/ERK-pathway genes in chemoresistant TNBC tumors after NAT.** Gene expression for SFK members (A, C) and members of the MAPK/ERK pathway (B, D) were determined at screening (NASC/Baseline) and after NAT (NA25/Surgery) in samples from the NeoAva cohort (A-B) and the GSE123845 dataset (C-D).

# Supplementary Fig. S9

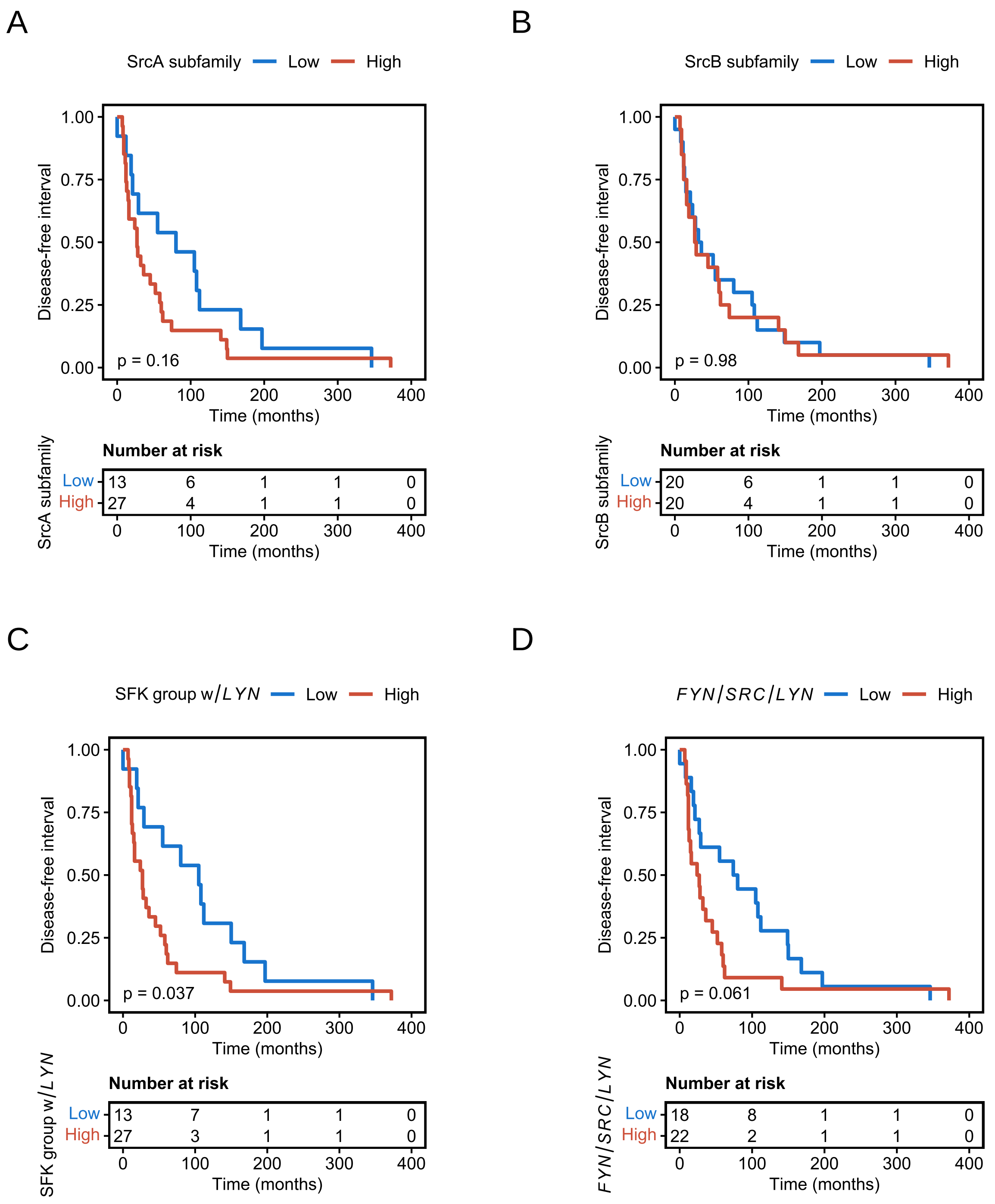


**Supplementary Figure S9. Expression of SRC-A sub-family members is associated with reduced disease-free interval in TNBC.** Disease-free interval for tumors from the MET500 cohort, where SFK *High* expression was defined as expression in >75% quantile for at least one SRC-A sub-family member (A), at least one SRC-B sub-family (B), including *LYN* in SFK score from Fig. 8C (C) or replacing *YES1* with *LYN (D)*.

# Supplementary Fig S10

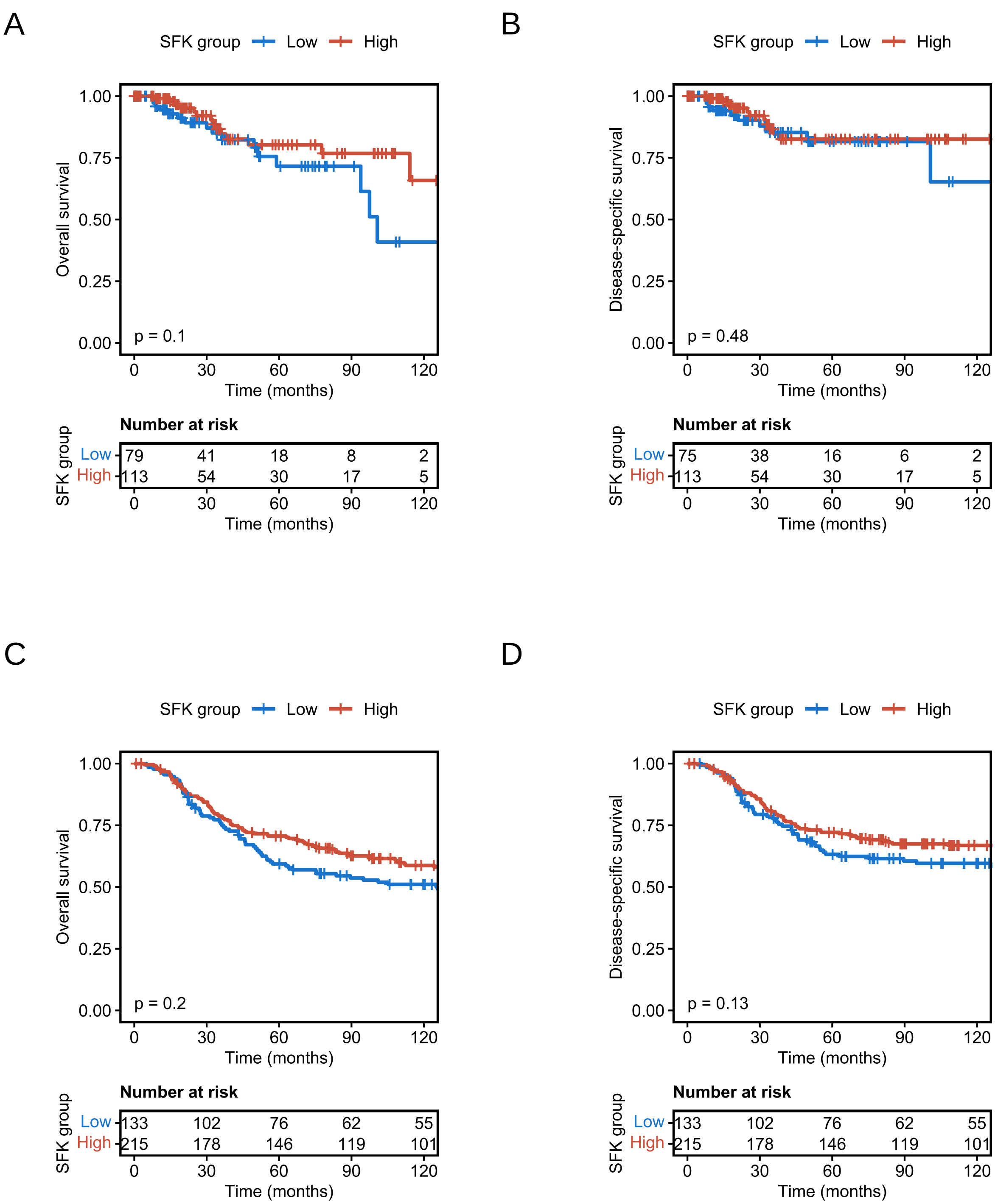


**Supplementary Figure S10. No association between expression of SFK members and survival in primary TNBC.** Overall survival (A, C) and disease-specific survival (B, D) was evaluated in TCGA (A-B) and METABRIC (C-D) cohorts, comparing *High* vs. *Low* SFK score as defined in Fig. 8C.

# Supplementary Fig S11 (Part 1 of 4)

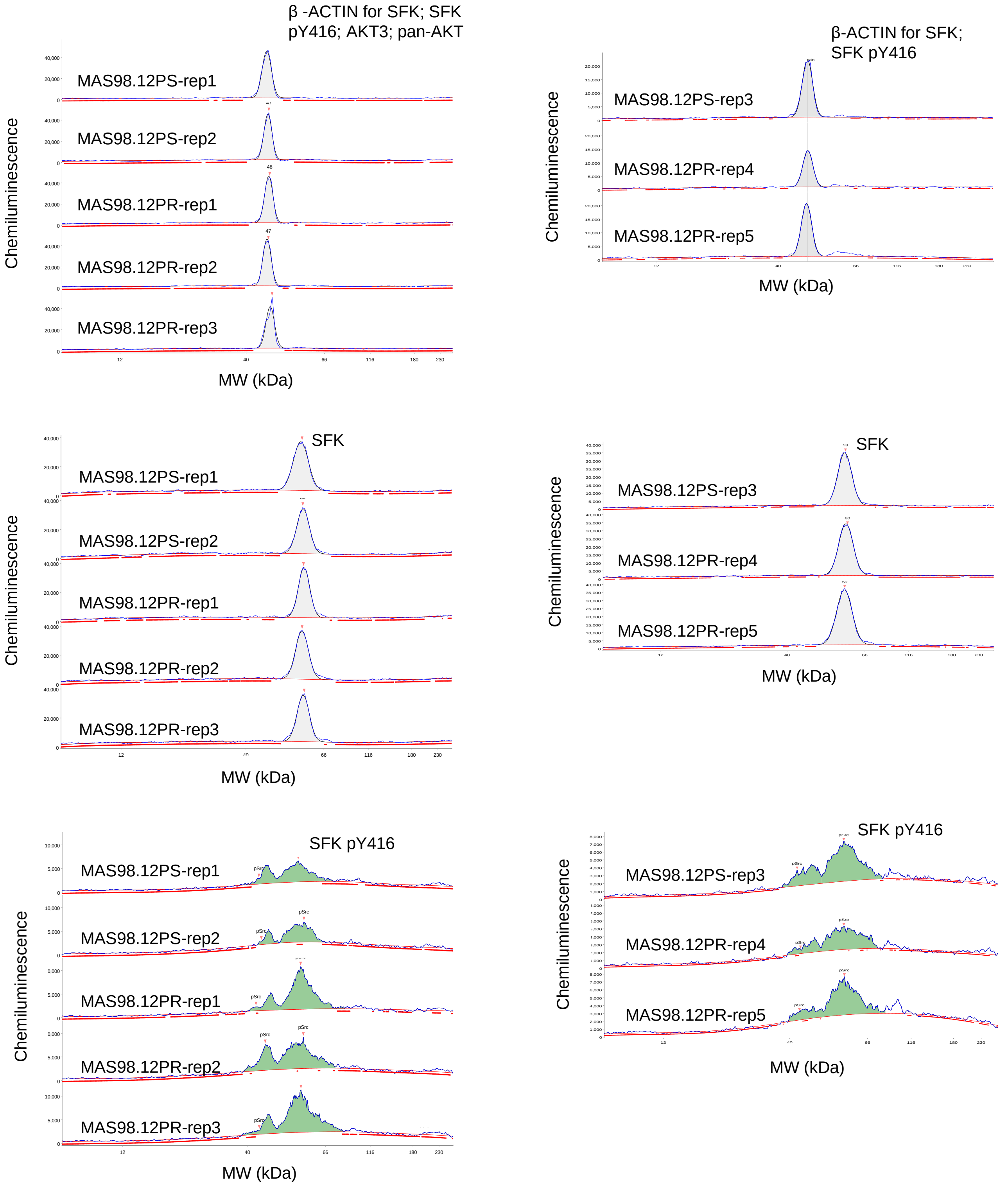


**Supplementary Figure S11. The peaks of indicated proteins detected by SWI that were used to calculate the protein expression levels presented in Figure 3B, E, H.** MAS98.12PS and MAS98.12PR PDXs were grown orthotopically before tumor collection and lysate preparation. Protein levels were calculated by integration of the area below peaks (colored area) detected by chemilumninescence. MAS98.12PS (n=3; rep1-3) and MAS98.12PR (n=5; rep1-5)

# Supplementary Fig S11 (Part 2 of 4)

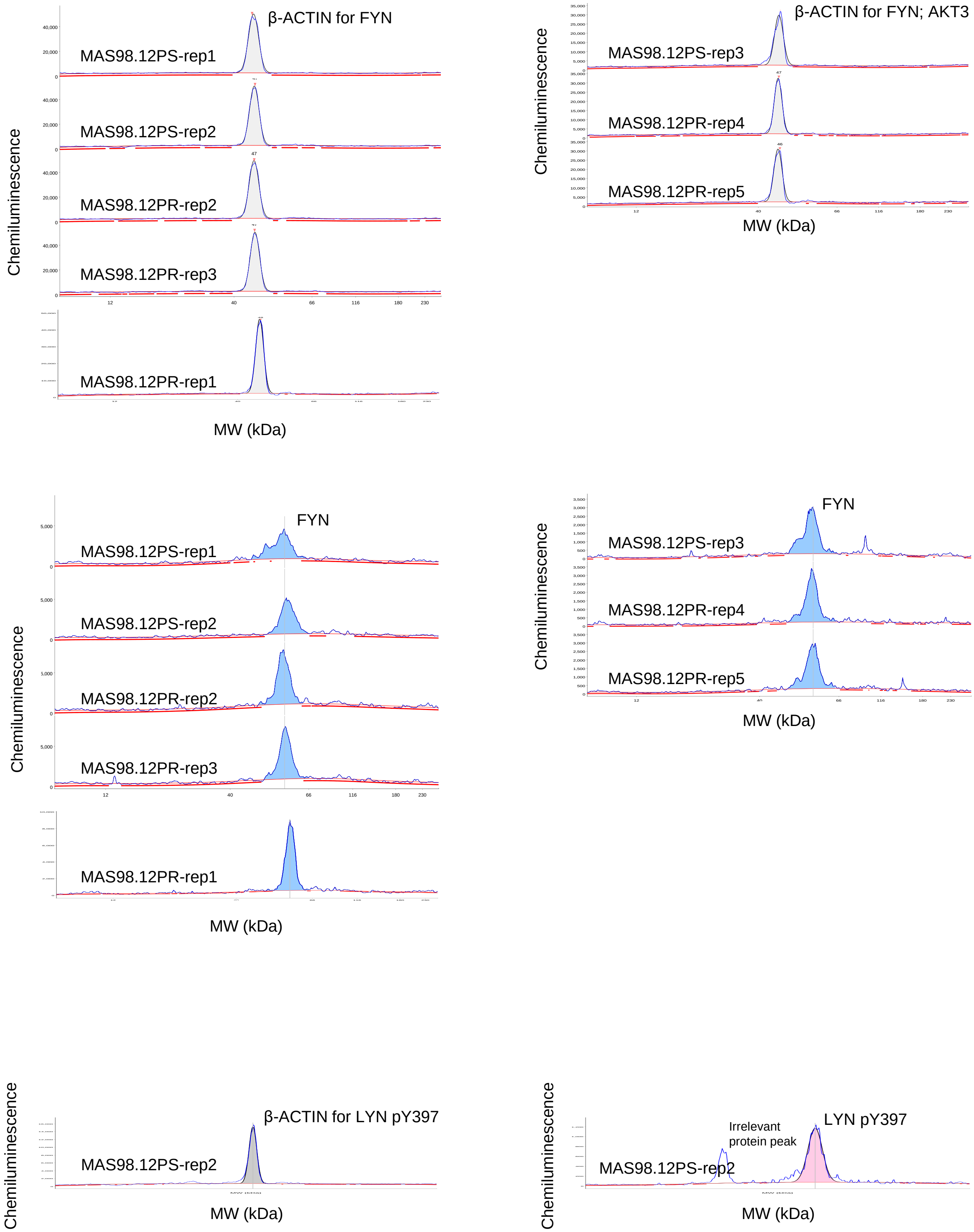

# Supplementary Fig S11 (Part 3 of 4)

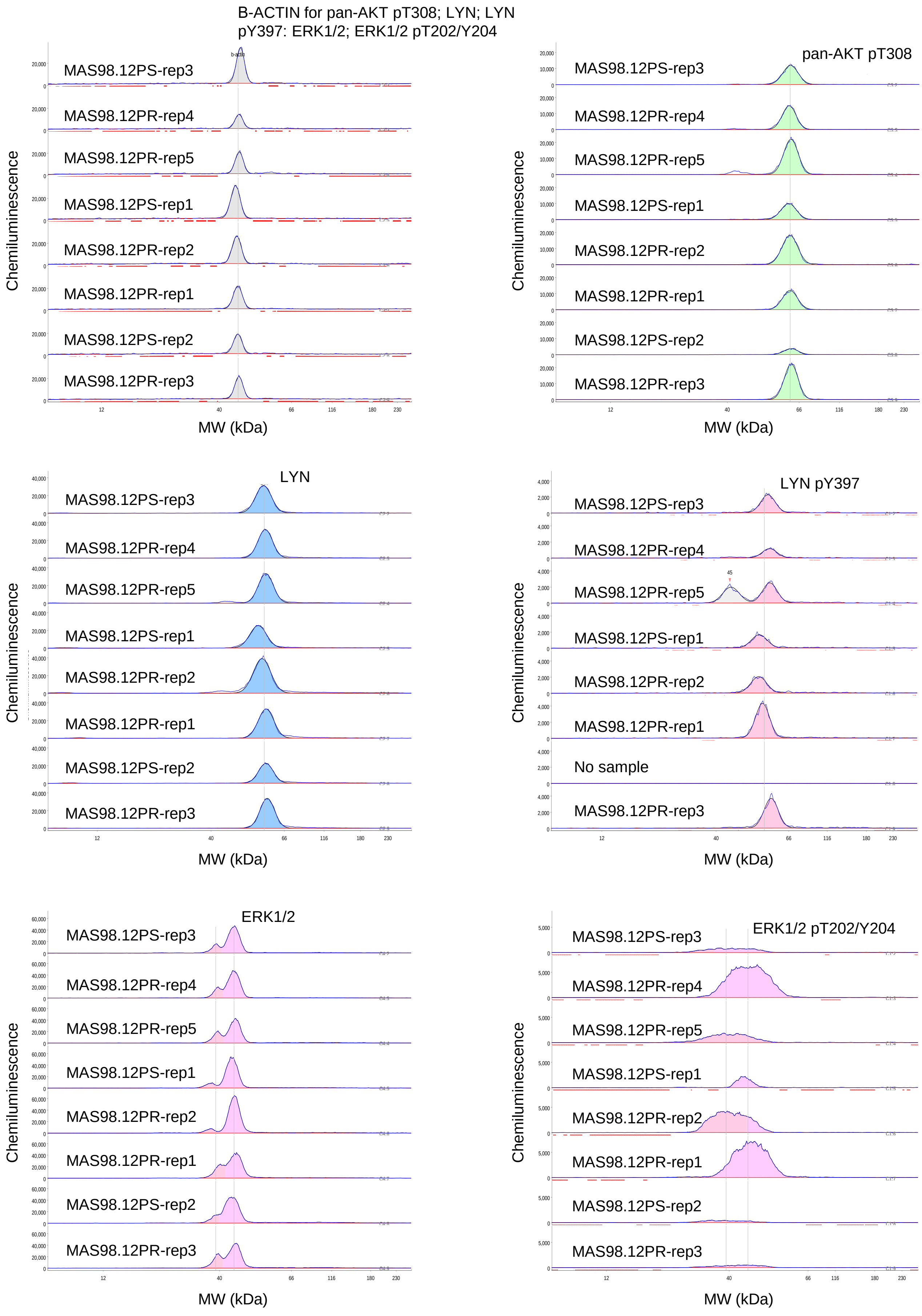

# Supplementary Fig S11 (Part 4 of 4)

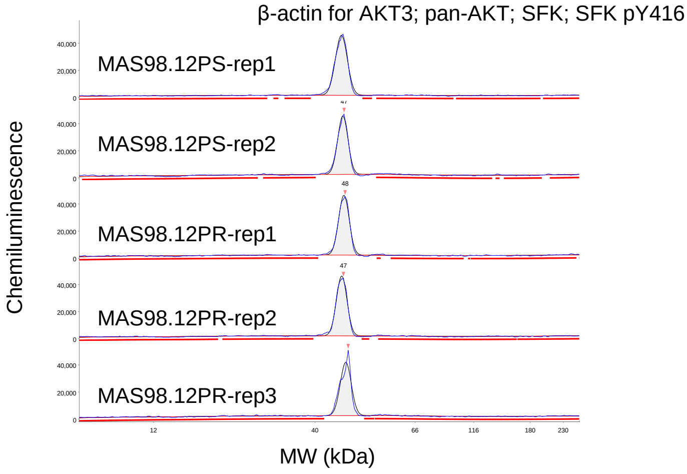


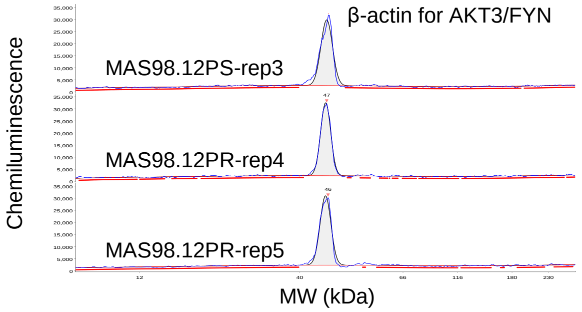


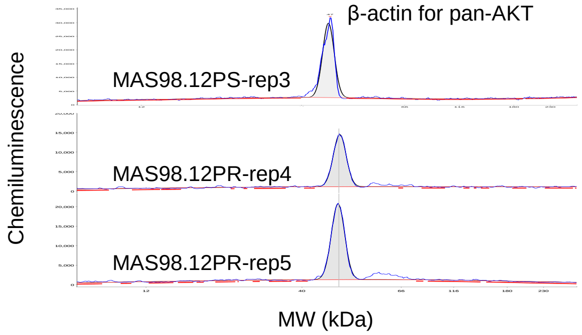


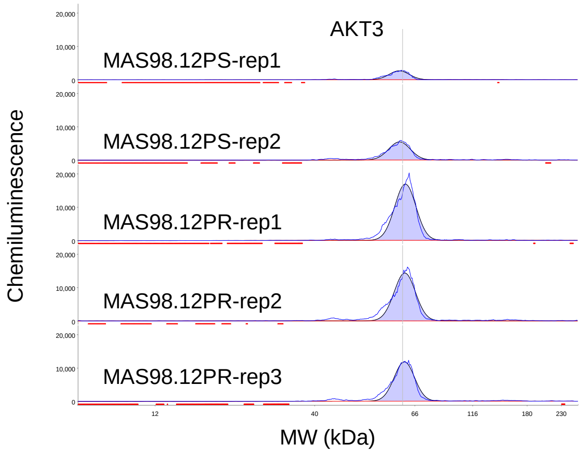


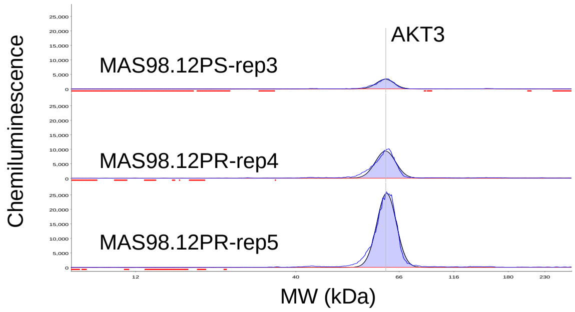


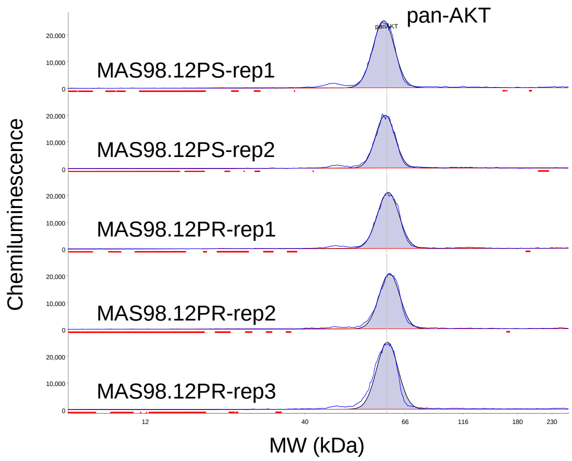


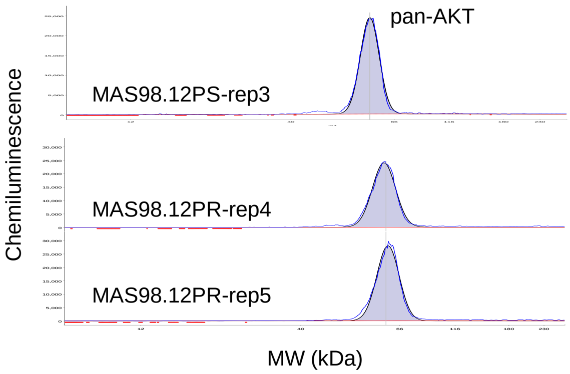

# Supplementary Fig S12 (Part 1 of 3)

A

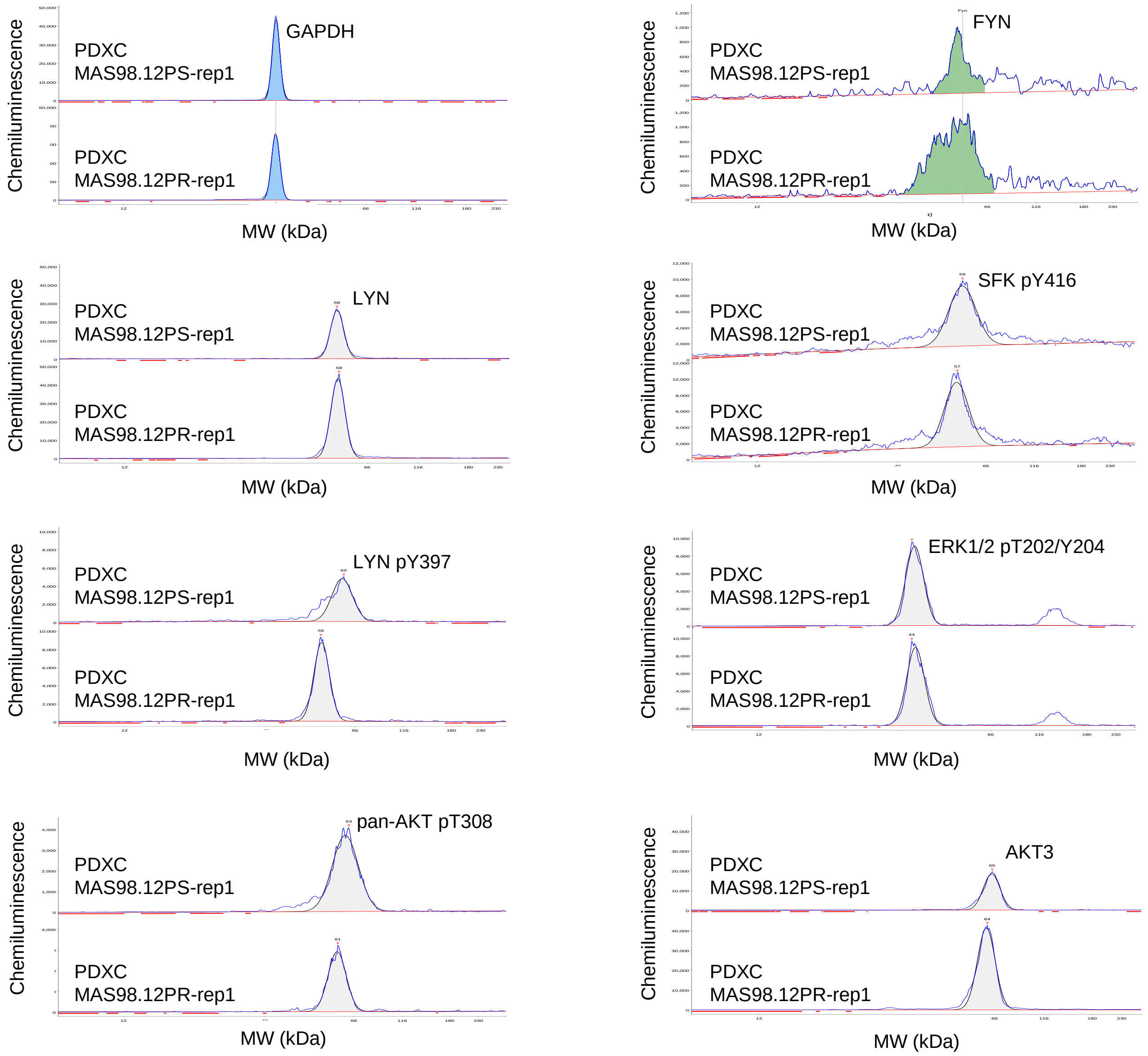


**Supplementary Figure S12. The peaks of indicated proteins detected by SWI that were used to calculate the protein expression levels presented in Figure S6B.** MAS98.12PS and MAS98.12PR PDXCs cultured *ex vivo* prior to sample collection and lysate preparation. Protein levels were calculated by integration of the area below peaks (colored area) detected by chemilumninescence. A-C indicates three distinct sets of SWI. MAS98.12PS (n=3; rep1-3) and MAS98.12PR (n=3; rep1-3).

# Supplementary Fig S12 (Part 2 of 3)

B

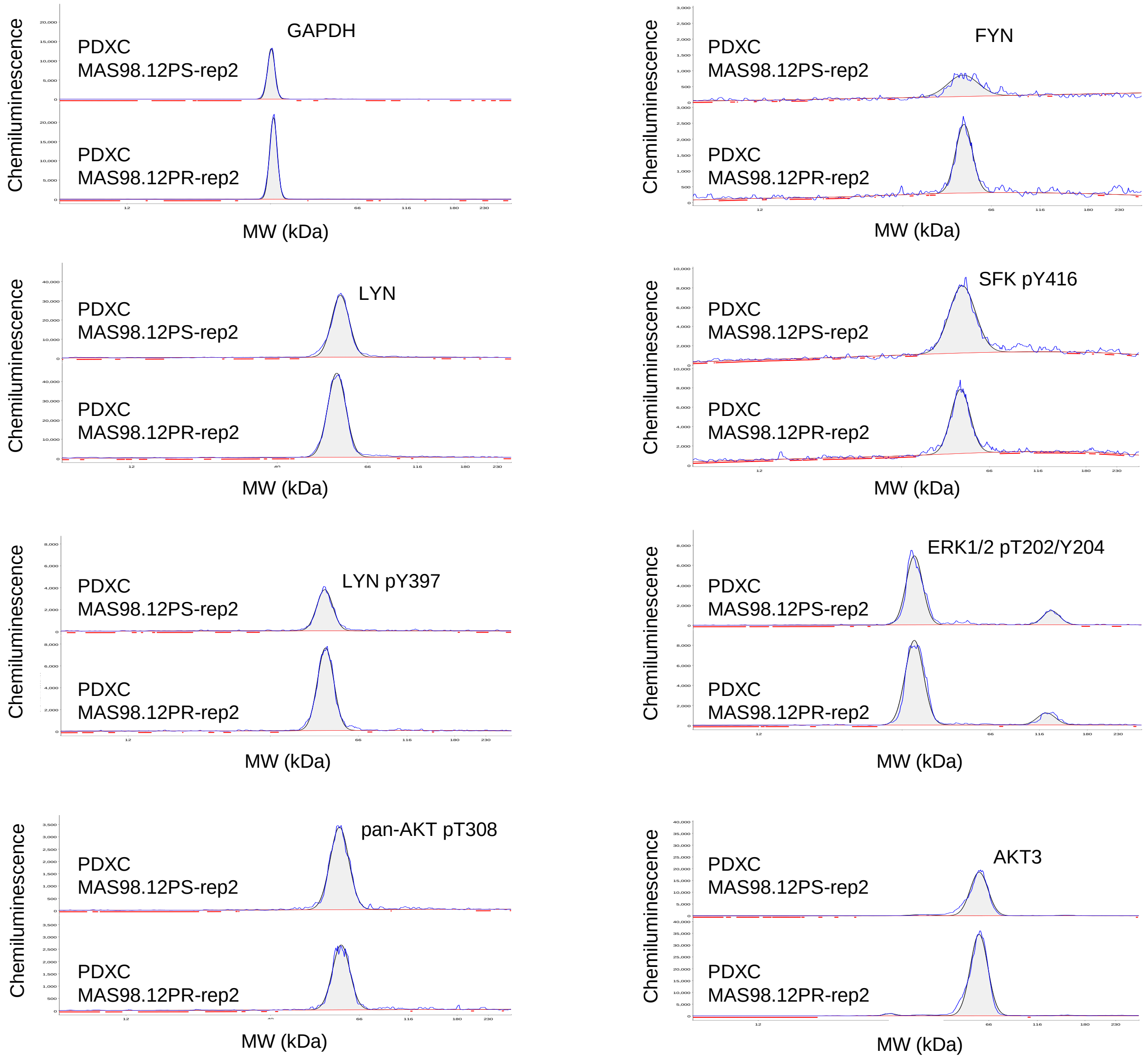

# Supplementary Fig S12 (Part 3 of 3)

C

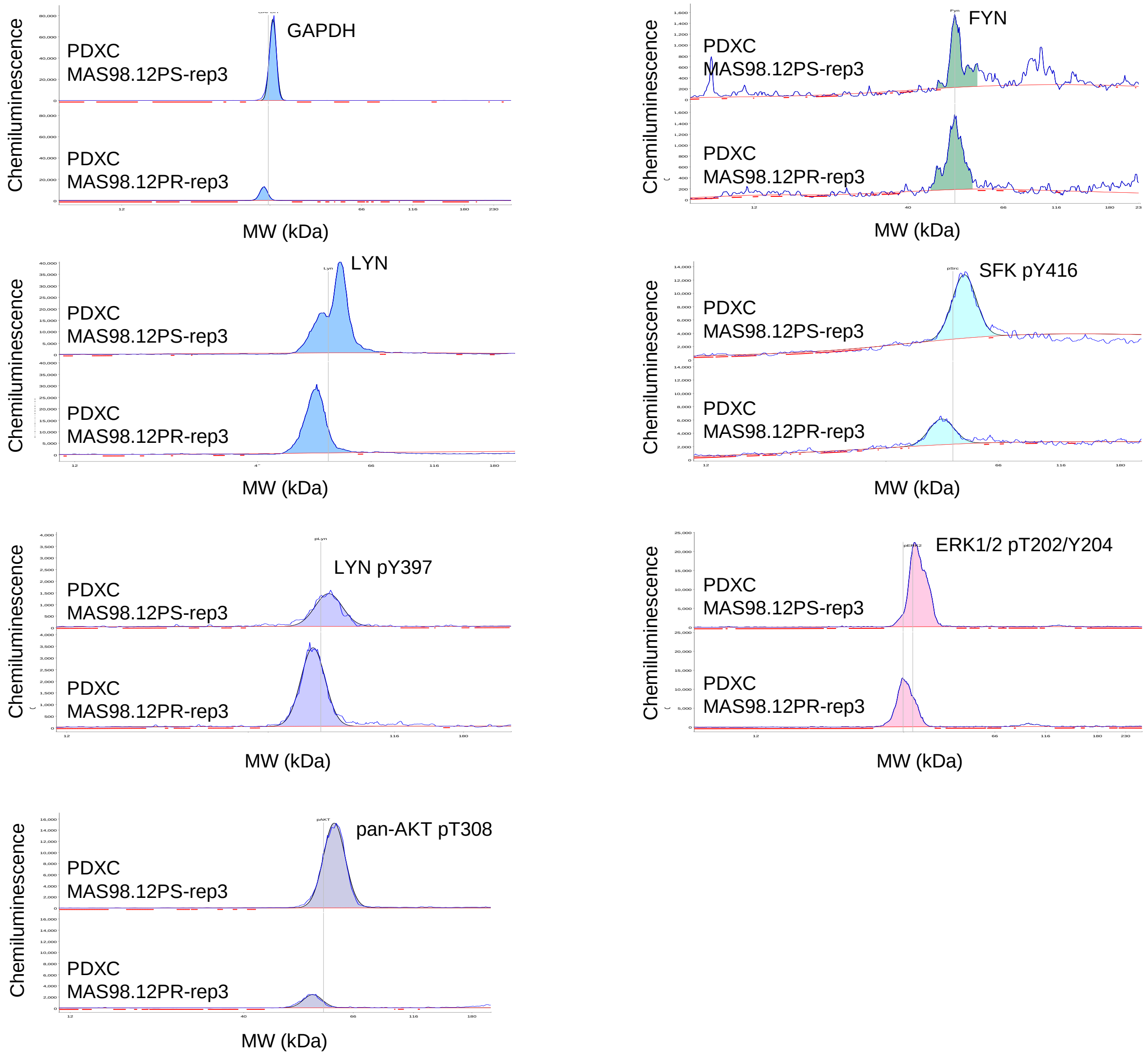

# Supplementary Fig S13 (Part 1 of 5)

A

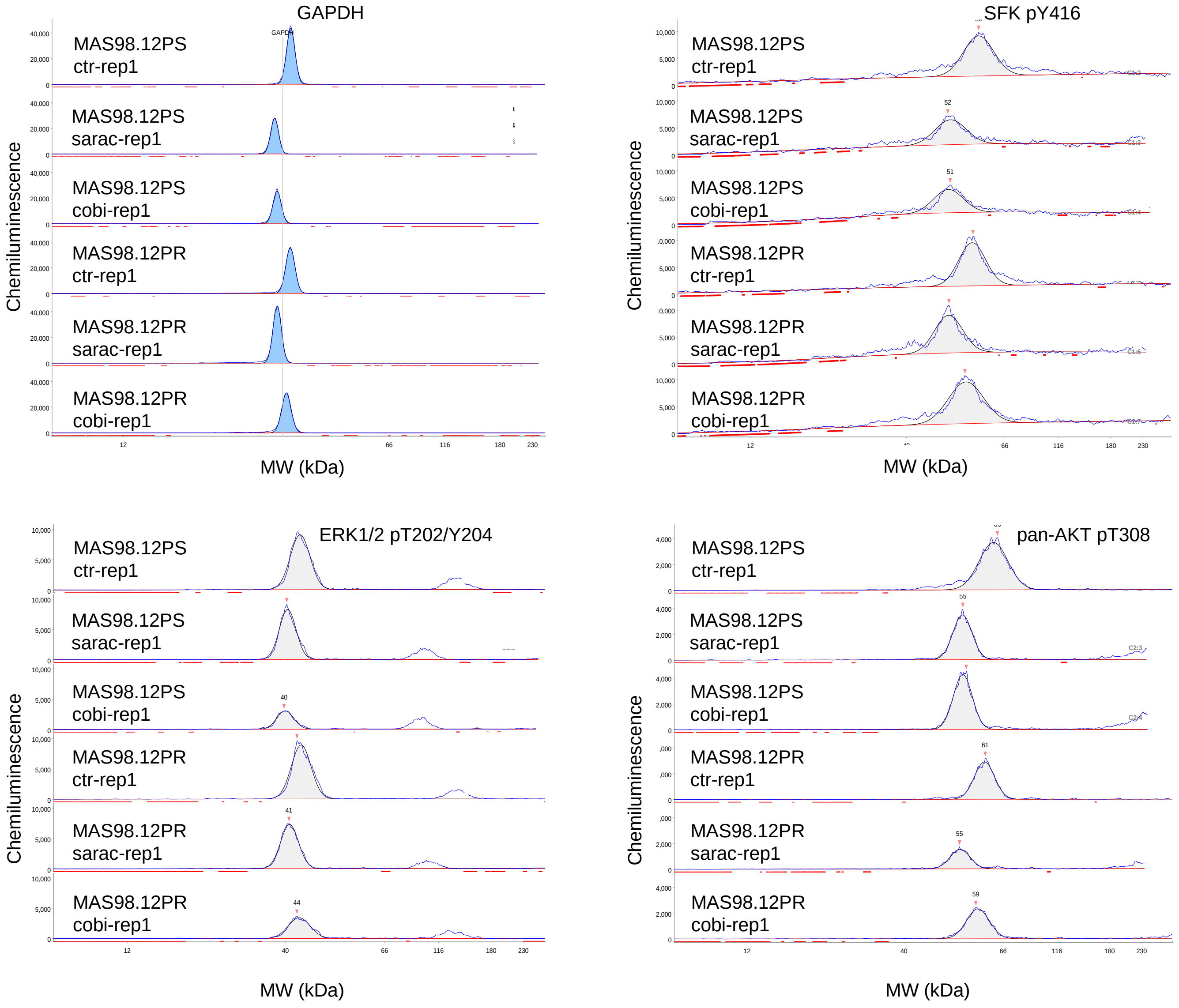


**Supplementary Figure S13. The peaks of indicated proteins detected by SWI that were used to calculate the protein expression levels presented in Supplementary Figure S7.** MAS98.12PS and MAS98.12PR PDXCs were cultured *ex vivo* and exposed to either 2 µM saracatinib (sarac) or 0.1 µM cobimetinib (cobi) for 3 hrs before sample collection and lysate preparation. Protein levels were calculated by integration of the area below peaks (colored area) detected by chemilumninescence. A-E indicates five distinct sets of SWI. MAS98.12PS (n=5; rep1-5) and MAS98.12PR (n=5; rep1-5).

# Supplementary Fig S13 (Part 2 of 5)

B

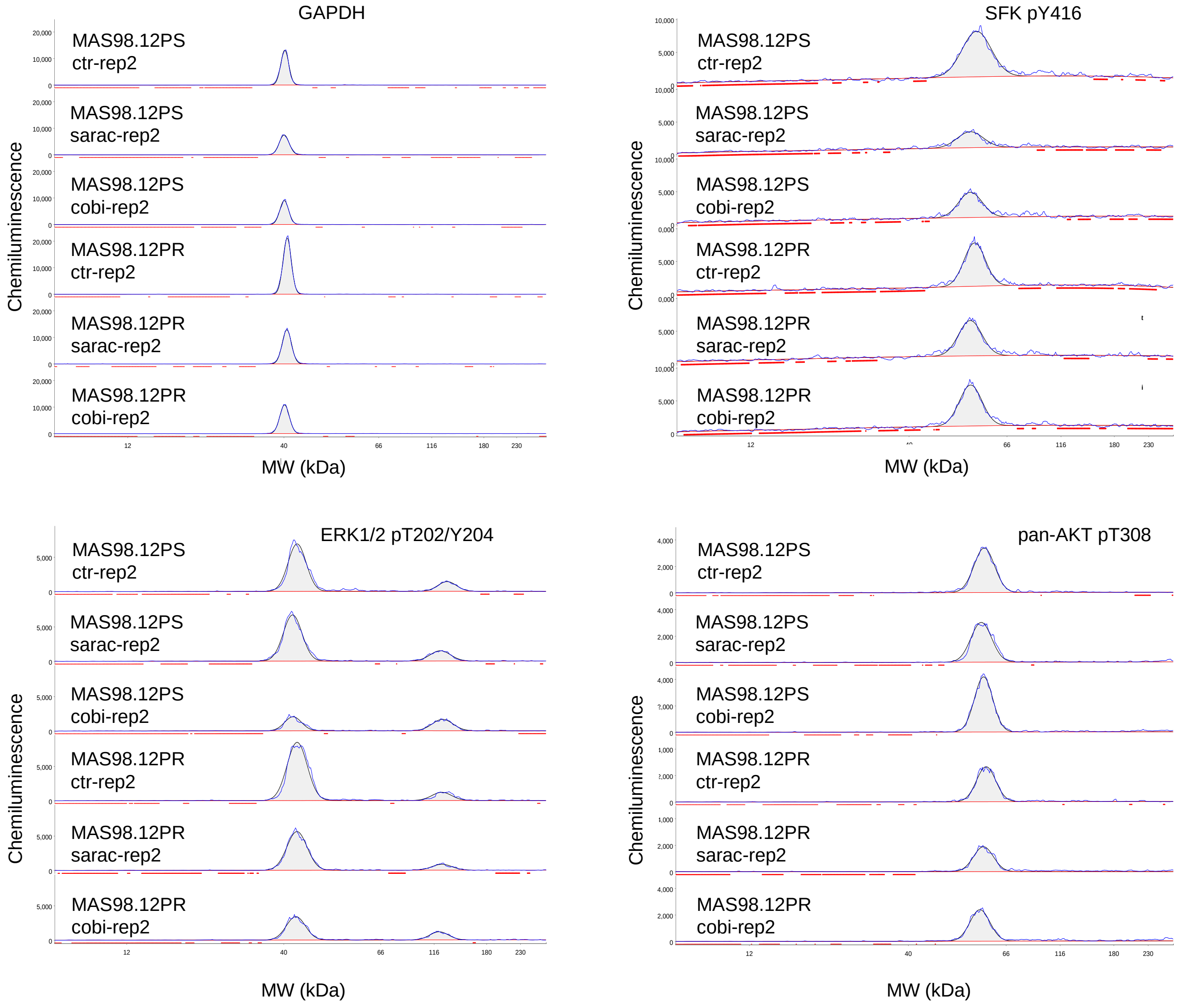

# Supplementary Fig S13 (Part 3 of 5)

C

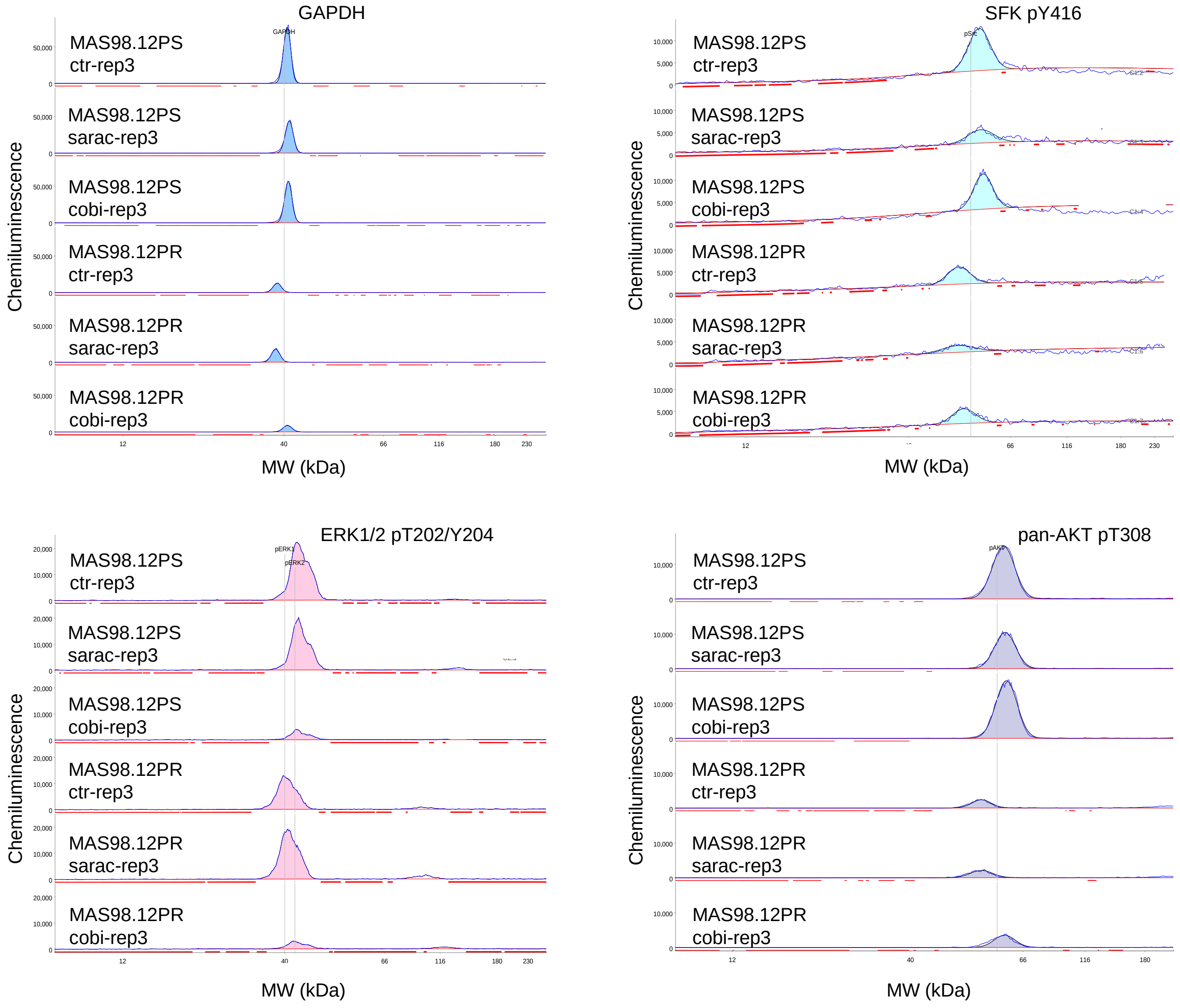

# Supplementary Fig S13 (Part 4 of 5)

D

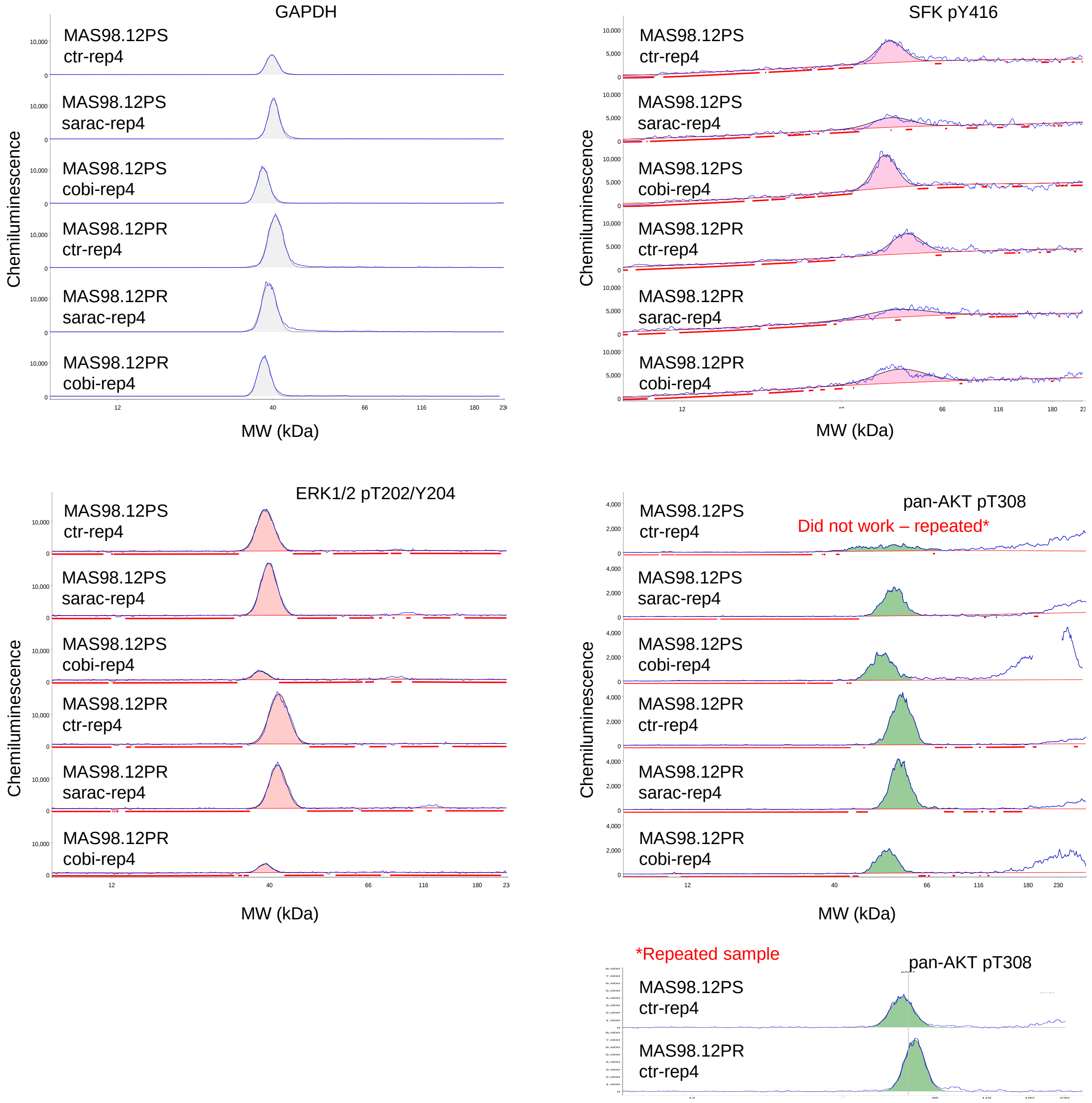

# Supplementary Fig S13 (Part 5 of 5)

E

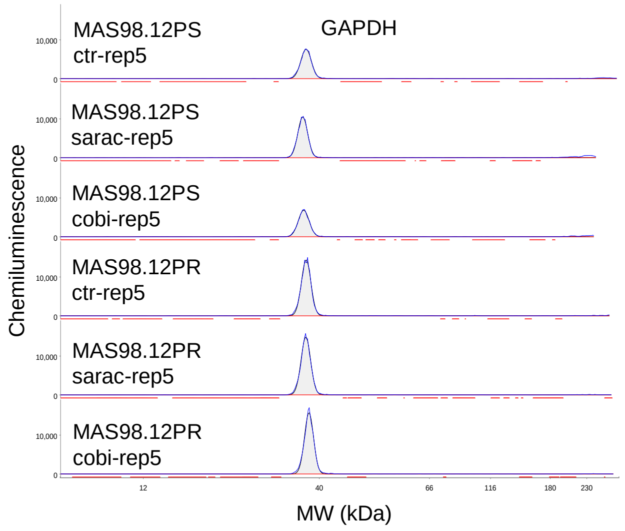


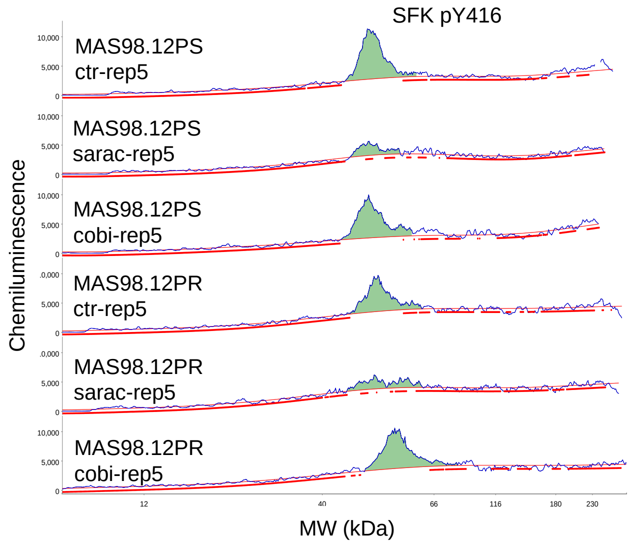


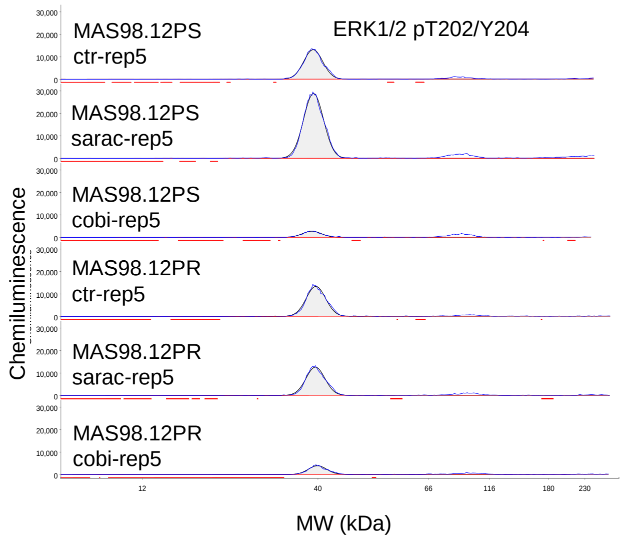


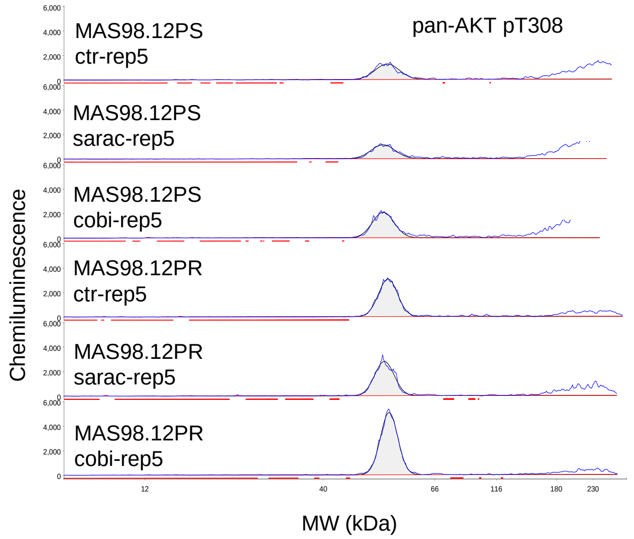

Supplement: Supplementary file 2 — Supplementary Figures [file 41416_2024_2875_MOESM2_ESM.pdf]
